# Human mining activity across the ages determines the genetic structure of modern brown trout (*Salmo trutta* L.) populations

**DOI:** 10.1111/eva.12266

**Published:** 2015-05-28

**Authors:** Josephine R Paris, R Andrew King, Jamie R Stevens

**Affiliations:** Biosciences, College of Life and Environmental Sciences, University of ExeterExeter, UK

**Keywords:** anthropogenic, DIYABC, genetic diversity, metal contamination, microsatellite, mining activity, population structure

## Abstract

Humans have exploited the earth's metal resources for thousands of years leaving behind a legacy of toxic metal contamination and poor water quality. The southwest of England provides a well-defined example, with a rich history of metal mining dating to the Bronze Age. Mine water washout continues to negatively impact water quality across the region where brown trout (*Salmo trutta* L.) populations exist in both metal-impacted and relatively clean rivers. We used microsatellites to assess the genetic impact of mining practices on trout populations in this region. Our analyses demonstrated that metal-impacted trout populations have low genetic diversity and have experienced severe population declines. Metal-river trout populations are genetically distinct from clean-river populations, and also from one another, despite being geographically proximate. Using approximate Bayesian computation (ABC), we dated the origins of these genetic patterns to periods of intensive mining activity. The historical split of contemporary metal-impacted populations from clean-river fish dated to the Medieval period. Moreover, we observed two distinct genetic populations of trout within a single catchment and dated their divergence to the Industrial Revolution. Our investigation thus provides an evaluation of contemporary population genetics in showing how human-altered landscapes can change the genetic makeup of a species.

## Introduction

The exploration for and exploitation of metals have played a crucial role in human history. Although metals are natural constituents of the earth's crust (Wedepohl 1991), their prevalence within natural systems has been significantly enhanced by human activity (Han et al. 2002). On a global basis, approximately half of all metal fluxes in the environment are anthropogenically driven (Wood 2011). Metals are extremely persistent in the environment; they are nondegradable, and thus, readily accumulate at toxic levels. Mining for such metals has a rich history in Britain. In particular, large areas of southwest England have been mined since the Bronze Age (2500 BCE: Dines 1956; Buckley 2002), with increasing exploitation during the Roman occupation (McFarlane et al. 2013), and later, as technology improved, throughout the Medieval period (Lewis 1965), with activity reaching its peak in the 19th century during the Industrial Revolution (Rainbow et al. 2011) when the region became the world's leading producer of many economically important metals (Dines 1956; Penhallurick 1986).

Natural populations are predicted to experience changes in genetic diversity and population structure, especially through genetic drift, gene flow and/or selection, but these genetic shifts have been shown to be amplified by anthropogenic interference (Banks et al. 2013)—for example, through habitat loss and fragmentation (Mondol et al. 2013); hunting pressure (González-Porter et al. 2011); overfishing (Allendorf et al. 2014); invasive species (Austin et al. 2011); and disease transmission (Kyle et al. 2014). Genetic data can provide a beneficial insight into this context. With respect to neutral markers, signatures of population-level disturbance may include reduced genetic diversity and associated population bottlenecks (Fontaine et al. 2012), alterations in population structure and disruption of gene flow (Clark et al. 2010), and signatures of loci under selection (Lind and Grahn 2011). The ability to quantify and understand these genetic processes is essential to informing conservation and management practices (Amos and Balmford 2001; Weeks et al. 2011).

Brown trout (*Salmo trutta* L.) populations are known to reside in rivers across southwest England, in both so-called clean and metal-impacted rivers. Due to the underlying geology (Webb et al. 1978) and ancient history of mining (Buckley 2002), metals are present in such ‘clean’ rivers, but at relatively low concentrations: River Camel (total zinc ∽17 μg/L; total copper ∽5 μg/L; total arsenic ∽4 μg/L); River Fal (total zinc ∽37 μg/L; total copper ∽5 μg/L; total arsenic ∽4 μg/L) (Environment Agency Data). On the other hand, metal rivers are defined by being heavily impacted by known historical mining activity. These rivers contain significantly elevated concentrations of metals: River Hayle (total zinc ∽350 μg/L; total copper ∽28 μg/L; total arsenic ∽9 μg/L); Red River (total zinc ∽238 μg/L; total copper ∽27 μg/L; total arsenic ∽86 μg/L) (Environment Agency Data). As these rivers are known to vary in their contamination levels, gradient effects between metal contaminant exposure and variation in genetic patterns of brown trout can be assessed.

Metal loads within such rivers can vary spatially over just a few kilometers (Environment Agency Data) meaning that genetic substructure may exist not only between, but also within a single system (Vähä et al. 2007). The River Hayle is a particularly well-studied metal-contaminated catchment. The whole river is affected by toxic metal pollution, dating back to the Industrial Revolution (19th Century), but is punctuated by an extremely high metal-contaminated middle region at Godolphin Cross (total zinc ∽2512 μg/L; total copper ∽417 μg/L; total arsenic ∽99 μg/L: Environment Agency Data). Metal contamination of such high levels is known to cause acute toxicity in metal-naïve brown trout (Hansen et al. 2006a,b; Durrant et al. 2011), yet brown trout exist along the river, both upstream and downstream of this middle region.

Using a panel of microsatellites, we sought to establish the patterns of genetic diversity and genetic structuring of trout from metal-impacted populations compared to trout from clean control rivers. Specifically, (i) Is genetic diversity reduced in metal-impacted populations?; (ii) Can these patterns be explained by genetic evidence of population bottlenecks?; (iii) Do metal-impacted populations show distinct genetic structuring due to reduced gene flow and genetic drift?; (iv) Given the long history of mining in the region, what is the most likely genetic origin of contemporary metal populations?; and finally, (v) Can the genetic changes we observe be linked to periods of increased mining activity? With a potential tie to human-mediated evolutionary change, analysis characterizing a temporal component to these genetic changes in a historical context is vital (Smith and Bernatchez 2008). To fully explore the spatial extent of mining practices on trout populations, we have taken a multiscaled approach, exploring evidence of genetic impacts across a larger region (southwest England) as well as conducting a microgeographic analysis of the River Hayle.

## Materials and methods

### Populations and sampling

**Figure 1:**
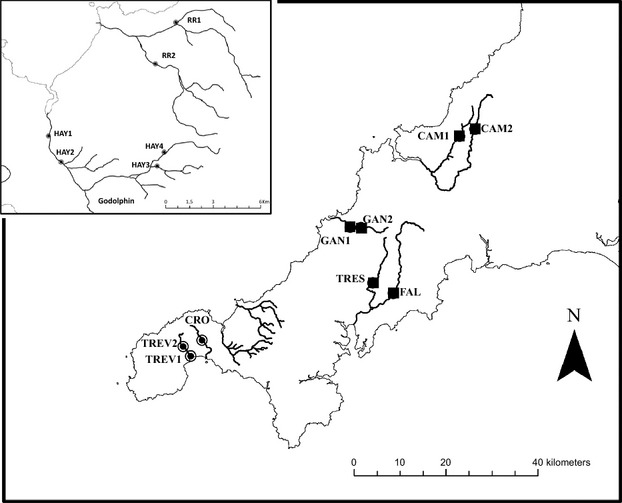
Geographic location of the populations sampled. Site codes correspond to those given in Table S1. Black squares represent clean sites, and black circles represent metal-contaminated sites. The enlarged map shows the sites on the Red River and the River Hayle.

A total of 700 individual brown trout (*Salmo trutta* L.) were included for study (Fig.1, Table S1). To account for within-river variation in metal-contamination levels, where possible, two sites per river were sampled. Of the 15 populations, six were selected as ‘clean’ control populations and the other nine were classified as originating from mining-impacted sites (Table S1). Brown trout sampled from each of these geographic sites will be referred to, and treated as populations. To the author's knowledge, there is no known history of supplementary stocking on the rivers included in this study.

Fish (1 + parr or older) were sampled as part of routine Environment Agency monitoring programs; briefly, fish were anesthetized using either clove oil or benzocaine (10 g/100 mL ethanol) diluted 1:2000 in river water prior to adipose fin clip removal. Fin clips were placed into 95% ethanol for storage.

### Microsatellite loci selection

Twenty-two putative neutral microsatellite loci were screened: Ssa85 and Ssa197 (O'Reilly et al. 1996); Ssa52NVH (Gharbi et al. 2006); SsosL417 and SsosL311 (Slettan et al. 1995); SS11 (Martinez et al. 1999); CA048828, BG935488, CA060208, CA060177, CA053293, CA515794 and CA769358 (Vasemägi et al. 2005); SsaD157 and SsaD58 (King et al. 2005); SsaF43 (Sánchez et al. 1996); Str3QUB (Keenan et al. 2013); Ssa407UOS and Ssa412UOS (Cairney et al. 2000); SSsp2213 (Paterson et al. 2004); One102 (Olsen et al. 2000 - using the primers of Keenan et al. 2013). Three MHC class 1-linked loci (sasaTAP2A, sasaTAP2B and sasa-UBA: Grimholt et al. 2002) were also chosen for screening due to their association with regions potentially under selection (Zelikoff 1993; Cohen 2002).

### DNA extraction, PCR, and genotyping

DNA was extracted from adipose fin clips using the HotSHOT method (Truett et al. 2000). PCR amplification was carried out using a BIO-RAD MyCycler Thermal Cycler in 10 *μ*L reaction volumes containing 1 *μ*L of extracted DNA (c. 30 ng DNA), 3 *μ*L RNase-free water, 5 *μ*L of QIAGEN HotStarTaq *Plus* Master Mix, and 1 *μ*L of primer mixture, in a total of 8 microsatellite multiplexes (Table S2). PCR conditions were as follows: an initial denaturing step at 95°C for 5 min was followed by 20 cycles of touchdown PCR consisting of 30 s at 94°C, a 30 s annealing step starting at 60°C or 55°C and decreasing by 0.5°C each cycle until a touchdown temperature of 50°C or 45°C (dependent on multiplex; Table S2) was achieved, and an elongation step of 72°C for 30 s, followed by 15 cycles comprising 94°C for 30 s, 50°C or 45°C for 30 s, and 72°C for 30 s. This was followed by a final 10 min extension step at 72°C. Genotyping was performed on a Beckman Coulter CEQ™ 8000 Genetic Analysis System.

### Full-sib analysis

To prevent biasing estimates of population genetic parameters, full-sibs within each population were identified using COLONY v 2.0.4.0 (Jones and Wang 2010). Each population was analyzed separately using a full-likelihood (FL) method, with a high-likelihood precision and a medium-length run. Two runs were performed under different random seeds. Any observed inconsistencies between these runs resulted in replicate runs being undertaken until the results were in consensus. Fish were considered members of a full-sib family if the probability of exclusion as full-sib families was >0.9. A single individual of each full-sib family was retained in the dataset.

### Data quality

The occurrence of homozygote excess, stuttering, large allele dropout, and null alleles were assessed using MICROCHECKER v 2.2 (van Oosterhout et al. 2004). Tests for linkage disequilibrium (LD) and Hardy–Weinberg equilibrium (HWE) were performed using GENEPOP v 4.2 (Rousset 2008), implementing the log likelihood ratio statistic and probability test, respectively. Default Markov chain parameters were used for both analyses. False discovery rate (FDR) was used to detect Type I errors in tests for LD and HWE (Storey and Tibshirani 2003).

### Genetic diversity

Three measures of genetic diversity were determined for each population. Allelic richness (*A*_R_) was calculated using FSTAT 2.9.3 (Goudet 1995) based on a minimum sample size of 26 diploid individuals. Observed heterozygosity (*H*_O_) and expected heterozygosity (*H*_E_) were calculated using GENALEX 6.5 (Peakall and Smouse 2012). Statistical differences in genetic diversity between population groups were calculated using FSTAT 2.9.3 (Goudet 1995), based on 1000 permutations of the dataset.

### Population bottlenecks

We used two approaches to test whether mining activity in the region has caused demographic declines in the studied trout populations. The program BOTTLENECK (Cornuet and Luikart 1996) uses the degree of heterozygosity excess compared to expectations under mutation–drift equilibrium to quantify the loss of rare alleles shortly after bottlenecks. Peery et al. (2012) have suggested that estimation of the number of multistep mutations at microsatellite loci is often underestimated and thus recommended a stepwise mutation rate of *p*_*S*_ = 0.78 (Peery et al. 2012). We therefore implemented the two-phase model (TPM) with 80% stepwise mutations (SMM). Deviations between observed and expected frequency distributions were statistically tested using Wilcoxon's signed-rank test for one-tail heterozygosity excess (*P *< 0.05). BOTTLENECK was run for 10 000 iterations. To further explore evidence of population bottlenecks, we also calculated the *M*-ratio, which is the ratio of the number of alleles to the range in allele size. This is expected to be lower in bottlenecked populations due to the loss of rare alleles (Garza and Williamson 2001). We used a prebottleneck effective population size (N_E_) between 50 and 100; theta (*θ*) thus varied from 0.1 to 0.2. We used a mutation rate of *μ *= 5 × 10^−4^ and used *p*_*g*_ = 0.22 (*p*_*S*_ = 0.78) and *δ*_*g*_ = 3.1, as suggested by Peery et al. (2012).

### Detecting loci under selection

We used three tests to detect whether any of the microsatellite loci showed evidence of selection. We used Bayescan v 2.1 (Foll and Gaggiotti 2008), which extends the distance-based method of Beaumont and Balding (2004) by adopting a Bayesian hierarchical model, implemented via reversible jump Markov chain Monte Carlo (RJ-MCMC) simulations. We used a burn-in of 50 000 iterations and a thinning interval of 10. Sample size was set at 5000, resulting in a total of 10^5^ iterations with 20 pilot runs (each with a length of 5000). We also used the statistic *lnRV* (Schlotterer 2002; Schlötterer and Dieringer 2005) to identify loci potentially hitchhiking with regions of the genome experiencing a selective sweep. We used the Bayesian two-way heterogeneous analysis of variance model (Marshall and Weiss 2006). Analyses were performed using WinBUGS v 1.4 (Lunn et al. 2000), implementing 20 000 MCMC iterations, with a 4000-iteration burn-in. Finally, we performed the Fdist test (Excoffier et al. 2009) in Arlequin v 3.5, which performs *F*_ST_ outlier simulations (Excoffier and Lischer 2010). We performed 3 separate runs of each dataset, each with 50 000 simulations under the hierarchical island model among groups of populations (*F*_CT_) with 100 demes simulated per group and 10 simulated groups. We considered a locus to be under selection if the *P*-value was <0.01.

### Population structure

Genetic differentiation was analyzed using global and pairwise *F*_ST_ estimates, calculated in GENALEX 6.5 (Peakall and Smouse 2012). Significance of estimates was based on 999 permutations of the dataset. *F*_ST_ was calculated for all loci in all populations, as well as independently for the River Hayle.

Genetic structuring of populations was analyzed using the model-based algorithm implemented in STRUCTURE v 2.3.3 (Pritchard et al. 2000), using a burn-in period of 50 000 iterations followed by 150 000 iterations with the number of inferred populations (*K*) ranging from 1 to 20. Ten independent runs of the program were performed using the population admixture model and correlated allele frequencies. The most likely number of population clusters was determined using the Δ*K* statistic (Evanno et al. 2005). To identify finer levels of structure, subsequent hierarchical analyses were performed based on the optimum *K* value from the first runs (Vähä et al. 2007). Analysis parameters for the hierarchical analyses were as given above except that maximum *K* was set at *n* + 2, where *n* represents the number of populations in the analysis.

To further explore population structure, a neighbor-joining dendrogram was constructed using the program POPULATIONS v1.2.28 (available at bioinformatics.org/∽tryphon/populations), based on the Cavalli-Sforza and Edwards chord distance, D_CE_ (Cavalli-Sforza and Edwards 1967). Statistical significance of branches was tested by bootstrap analysis based on 1000 iterations. The dendrogram was visualized in MEGA v 6 (Tamura et al. 2013).

### Placing time points on genetic divergence

We used approximate Bayesian computation (ABC) as implemented in the program DIYABC (Cornuet et al. 2008, 2014) to explore the genetic history of the contemporary trout populations, divergence times between these populations, and also to determine changes in effective population size (N_E_). DIYABC outputs averages for each event in generations. We converted generations into dates using a generation time for brown trout of 4 years (Jensen et al. 2008).

#### Preliminary investigations

Based on the results of the population structure analyses of this study, we conducted preliminary investigations based on simple scenarios comparing the general clean group to each metal group: (i) Clean & Hayle, (ii) Clean & Red River, and (iii) Clean & Crowlas/Trevaylor. For the River Hayle analyses, we specified two time (t)-splitting points, t1: the split within the River Hayle and t2: the split of the Hayle populations from the clean populations. Conditions were set so that t2 > t1. For the Red River and Crowlas/Trevaylor investigations, only one time-splitting point was specified (t1), defining the split between the metal-impacted group and the clean group. For each of these independent population comparisons, we passed two scenarios, each composed of a reference table consisting of 10^5^ simulated datasets. Scenario 2 differed from Scenario 1 by the inclusion of a reduction in population size following the split between the clean and metal populations, as suggested by genetic diversity estimates calculated here (see Results). Default minimum and maximum priors (10–10 000) were used for all parameters (N—effective population size; Nb—prebottleneck effective population size; t—time-splitting point(s); db—duration of bottleneck).

#### Hypotheses-testing scenarios

Based on the outcome of the preliminary investigations, we constructed more complicated scenarios that included genotypes from all of the populations, organized into 5 population groups: (i) clean populations; (ii) RR1&2 (Red River); (iii) CRO/TREV1&2 (Crowlas and Trevaylor); (iv) HAY1&2 (downstream Hayle); and (v) HAY3&4 (upstream Hayle). We constructed 3 groups of hypotheses-testing scenarios, the details of the topology of these can be found in Fig. S1. Group 1 consisted of scenarios by which the clean and metal populations are independently derived from a common ancestor that is neither definitely clean nor metal in its genetic background. Group 2 scenarios tested variations on the hypothesis that metal populations are derived multiple times from a clean lineage. Group 3 comprised scenarios where the clean and metal groups are separate lineages, with metal populations being derived from the common metal lineage. Based on observations of preliminary runs, the maximum prior for the t1 parameter (Hayle split) was set at 3000 generations (12 000 years). The duration of bottleneck (db) was set at a maximum of 300 generations (1200 years). All other parameter priors remained as default values (10–10 000). Conditions were placed on splitting time points so that t4 > t3; t3 > t2; and t2 > t1.

#### Summary statistics and model checks

For all investigations and scenarios, we used the summary statistics of Cornuet et al. (2008): One-sample summary statistics included mean number of alleles; mean genic diversity; mean Garza-Williamson's *M,* and two-sample summary statistics included *F*_ST_ and mean classification index. We used the default priors for the mutation model (Min: 10^−4^; Max: 10^−3^; Mean: 5 × 10^−4^). We performed model checking using a PCoA, and posterior probabilities of each scenario were then calculated using a logistic regression of 1% of the simulated data. After bias and precision estimations, model checks were performed for each scenario, using the summary statistics of Guillemaud et al. (2010), which include the two population summary statistics: mean number of alleles, mean genic diversity, mean size variance, *F*_ST._ and the classification index. For the group-based hypotheses scenarios, we simulated 10^5^ runs for each scenario, after which we selected the scenario(s) from each group with the highest posterior probability. These scenarios were then compared in a final run, simulating 10^6^ datasets per scenario, using the same priors, summary statistics, and model checks as outlined above.

## Results

### Data quality and correction

A total of 700 individuals from 15 brown trout populations were genotyped. Fifty-nine individuals (∽8.4%) were removed due to sibling effects within the sampling. The 24 primer sets amplified a total of 25 loci, with the primers for One102 amplifying two loci with nonoverlapping size ranges (designated One102a and One102b). There were potential genotyping errors at 14 loci. We removed locus CA053293, as these inaccuracies occurred in 7 of the 15 populations. Evidence of homozygote excess and null alleles was not consistently detected in any other loci.

Indication of linkage disequilibrium (LD) between sasa-UBA & sasaTAP2B was statistically significant in 12 of the 15 populations. There is evidence that these loci are in fact physically linked (Grimholt et al. 2002). Due to difficulty in scoring, it was decided to omit locus sasa-UBA from further analysis. Tests for Hardy–Weinberg equilibrium (HWE) revealed that five loci (Ssa412UOS, Ssa407UOS, Ssa52NVH, SsaD157, and SsosL417) showed significant deviation from HWE. However, as no locus was considered to be out of HWE in more than one population, all loci were retained in subsequent analyses.

### Genetic diversity

Metal-impacted populations had lower genetic diversity in comparison with populations from the clean sites (Table1). These differences were statistically significant (*A*_R_, *P* = 0.001; *H*_E_, *P* = 0.001; *H*_O_, *P* = 0.002). Allelic richness (*A*_R_) varied between 6.2 (HAY1) and 11.15 (CAM2). Similar patterns were observed for measures of heterozygosity: Expected heterozygosity (*H*_E_) varied from 0.61 (HAY2) to 0.78 (CAM2 & FAL) and observed heterozygosity (*H*_O_) ranged between 0.61 (HAY2) and 0.79 (CAM2). In particular, genetic diversity measures showed marked differences between the River Hayle and the River Camel, the mouths of which both flow out of north Cornwall; approximately 55 km from one another.

**Table 1:** Measures of population genetic parameters for each population using 23 microsatellite loci

Population	*N*_1_	*N*_2_	*A*_R_	*H*_E_	*H*_O_	Wilcoxon TPM	M-ratio
CAM1	49	47	9.89	0.77	0.75	ns	0.59
CAM2	44	44	11.15	0.78	0.79	0.003	0.61
GAN1	50	49	9.51	0.75	0.76	ns	0.56
GAN2	50	45	8.92	0.74	0.73	0.052	0.58
FAL	47	42	10.25	0.78	0.77	ns	0.61
TRES	48	46	10.01	0.76	0.75	ns	0.60
RR1	45	41	6.87	0.70	0.70	0.000	0.50
RR2	41	40	7.79	0.70	0.69	0.008	0.57
HAY1	44	43	6.20	0.62	0.62	0.015	0.47
HAY2	48	39	6.23	0.61	0.61	0.006	0.49
HAY3	48	42	6.68	0.65	0.65	0.019	0.50
HAY4	37	27	6.34	0.63	0.64	ns	0.50
CRO	49	46	6.96	0.65	0.68	0.011	0.51
TREV1	50	45	9.36	0.74	0.74	ns	0.58
TREV2	50	45	7.30	0.70	0.70	ns	0.52

*N*_1_—number of sampled individuals, *N*_2_—number of individuals after full-sib removal, *A*_R_ —allelic richness, *H*_E_—expected heterozygosity, *H*_O_—observed heterozygosity, (i) Wilcoxon one-tail test results from BOTTLENECK, (ii) average *M* from *M*-ratio.

Across the metal populations, TREV1 had unusually high levels of genetic diversity, more similar to levels observed in clean populations. The River Hayle had very low genetic diversity when compared not only to the clean populations (*A*_R_ = 0.005; *H*_E_ = 0.001; *H*_O_ = 0.001) but also to other metal-impacted populations (*A*_R_ = 0.005; *H*_E_ = 0.08, *H*_O_ = 0.04). Sites downstream of the Godolphin region (HAY1, HAY2) had lower genetic diversity, compared to the two sites upstream (HAY3, HAY4), although these differences were not statistically significant. In particular, there was a marked difference in genetic diversity between the two sites straddling the metal region, which are separated by just 8 km; the upstream site (HAY3) had the highest genetic diversity (for *A*_R_, *H*_E_, *H*_O_), whereas the site immediately downstream (HAY2) had the lowest genetic diversity (for *H*_E_ & *H*_O_).

### Population bottlenecks

With the BOTTLENECK program, the Wilcoxon one-tailed test for heterozygosity excess was significant for three of the Hayle sites (HAY1, HAY2, and HAY3), and also for other metal populations (RR1, RR2, and CRO) (Table1). This suggests that these metal-impacted populations have experienced recent population declines. There was also evidence that the clean CAM2 and GAN2 populations have also bottlenecked, although these populations exhibited high overall genetic diversity.

*M*-ratio values suggested that bottlenecks had occurred in all populations—both metal-impacted and clean (Table1). Across all sites and loci, the *M*-ratio ranged from 0.47 to 0.61, which in every case was lower than the calculated critical value (0.87). Furthermore, these values were also lower than the critical value of 0.68 proposed by Garza and Williamson (2001). This method did however seem to reflect the influence of metal contamination, as trout from metal-impacted sites had lower *M*-ratios compared to trout from clean sites (average 0.51 and 0.6, respectively).

### Loci under selection

Tests for loci under selection using the Bayescan approach of Foll and Gaggiotti (2008), the *lnRV* statistic (Marshall and Weiss 2006), and the Fdist method (Excoffier et al. 2009) showed no reliably identifiable signals of diversifying selection in the various population group analyses (Fig. S2). Except for a signature of diversifying selection in the Red River populations for sasaTAP2A using the *lnRV* statistic, the MHC-linked loci showed little evidence of positive selection.

### Genetic differentiation and population structure

Global *F*_ST_ for all populations and loci was 0.098. The highest *F*_ST_ between all populations was between HAY2 and CRO (*F*_ST_ = 0.106, *P* = 0.001, Table S3). The lowest *F*_ST_ was between the two downstream Hayle sites, HAY1 and HAY2 (*F*_ST_ = 0.006, *P* = 0.6). All pairwise *F*_ST_ values were statistically significant (*P* < 0.05), except for HAY1 and HAY2. Within the River Hayle, the global *F*_ST_ was 0.031. The highest *F*_ST_ was between the HAY2 and HAY4 sites, *F*_ST_ = 0.029 (*P* = 0.001), and the lowest was between HAY1 and HAY2, *F*_ST_ = 0.006 (*P* = 0.622). Across the Godolphin middle region, the pairwise *F*_ST_ between the downstream and upstream Hayle populations was 0.021 (*P* = 0.001).

**Figure 2:**
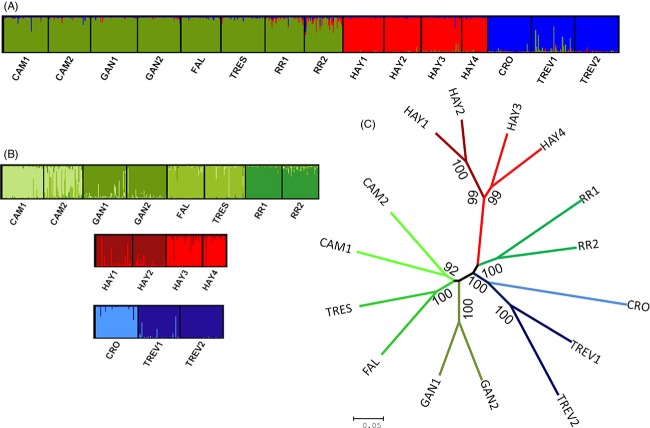
(A and B) Hierarchical STRUCTURE analyses showing estimated proportions of the coefficient of admixture of each individual's genome that originated from population *K*. (A) Primary STRUCTURE plot of all populations (*K *=* *3). (B) From left to right; in green: hierarchical plot, where *K *=* *4; in red: hierarchical plot, where *K *=* *2; in blue: hierarchical plot, where *K *=* *3. (C) Neighbor-joining phenogram, based on Cavalli-Sforza and Edwards chord distance (*D*_CE_) showing the relationships between 15 populations of brown trout. Bootstrap values (% based on 1000 replicates) are show next to relevant branches; only bootstraps >90% are shown in the figure. Colors for each population match those presented in A and B.

Analysis of population structure using STRUCTURE showed that the most likely number of genetic groups was *K *=* *3 (Fig.2a; Δ*K* = 464.96): Group 1 (green) comprised CAM1, CAM2, GAN1, GAN2, FAL, TRES, RR1, and RR2; Group 2 (red) comprised the four Hayle populations (HAY1-HAY4); and Group 3 (blue) included the CRO, TREV1, and TREV2 populations (Fig.2A). For Group 1, the hierarchical analyses showed Δ*K* = 3, whereby the groups were differentiated based on the river basin of origin, so that CAM1 & CAM2; GAN1 & GAN2; FAL & TRES, and RR1 & RR2 grouped together (Fig.2B). Hierarchical analysis of Group 2 showed differentiation between the populations upstream and downstream of the highly contaminated Godolphin region of the Hayle (Fig.2B). For Group 3, the hierarchical analysis separated the Crowlas from the two populations originating from the Trevaylor (Fig2B).

The neighbor-joining dendrogram (Fig.2C) revealed four population groups, each with high bootstrap support. The first group comprised the clean populations. Within this group, further structure was supported with high bootstraps, defining the population groups by river basin. The second group comprised the Hayle populations (HAY1-HAY4). The split between the upper and lower reaches of the Hayle was also evident. The third group includes the two sites from the Red River (RR1 & RR2), which cluster most closely to the Hayle but are still genetically distinct, despite their close geographical proximity. The final group forms the other three metal-impacted populations: the Crowlas (CRO), and the two sites on the Trevaylor (TREV1 & TREV2).

### Dating genetic divergence

For the preliminary investigations (see Materials and methods), all population-group comparisons showed that the posterior probability of Scenario 2 was higher, and therefore, it was more likely that population bottlenecks occurred prior to the generation of each metal-impacted population. This is further confirmed by the measures of genetic diversity (Table1). Four scenarios were included in the final analysis (Fig. S1)—one scenario each from Groups 1 and 2 and two scenarios from Group 3 (as both scenarios had high logistic regression values). The most likely scenario was where metal-affected populations were derived multiple, independent times from a general clean lineage (logistic regression = 0.9721: Fig. S1: Group 2, Scenario 1). The mean mutation rate was 5.34 × 10^−4^ (95% CI: 3.23 × 10^−4^ to 8.71 × 10^−4^). Using a generation time of 4 years, this split occurred approximately 960 years ago, c.1050 AD (t2 = 240 generations, 95% CI: 96.5–730; Table2). After the split, the effective population size (N_E_) of the Red River populations reduced by ∽34% and the Trevaylor and Crowlas populations actually showed a small (∽4%) increase in N_E_. The subsequent split within the Hayle (t1) occurred approximately 155 years ago, c.1860 AD (t1 = 38.9 generations, 95% CI: 15.3–156). The River Hayle populations experienced a ∽70% reduction in N_E_. This reduction is the most severe of all population declines calculated (Table2).

**Table 2:** Median values and 95% confidence intervals (CI) for DIYABC parameters for Scenario 1, Group 2 (See Fig. S1 for scenario topography).Values are in generations

Parameter	Median	95% CI
N_clean_	9220	7670–9930
N_Red river_	3270	1430–6870
N_Crowlas,Trevaylor_	5640	2960–8680
N_Hayle Downstream_	1400	605–3590
N_Hayle Upstream_	2900	1100–6900
t1_Hayle split_	38.9	15.3–156
t2_Clean-metal split_	240	96.5–730
DB_Red river_	165	27.2–288
N2_Red river_	4950	588–9480
DB_Crowlas,Trevaylor_	159	24.9–286
N2_Crowlas,Trevaylor_	5430	748–9570
DB_Hayle_	180	29.6–290
N2_Hayle_	4570	485–9430
*μ*mic	5.34 × 10^−4^	3.23 × 10^−4^ to 8.71 × 10^−4^

N = effective population size after bottleneck, N2 = effective population size before bottleneck, DB = duration of bottleneck. *μ*mic = mean mutation rate.

## Discussion

The question of whether the genetic patterns of natural populations can be altered by human disturbance is a salient issue in modern ecology and conservation. We used a panel of microsatellites to investigate the capacity of metal mining to act as a driver of genetic change in brown trout populations, on both a local and a regional scale. We explored both the genetic diversity and differentiation of trout occupying metal-impacted rivers compared to trout from relatively unpolluted control sites. Using approximate Bayesian computation (ABC), we have placed time points on the demographical changes of these populations to elucidate how present-day genetic patterns have been affected by historical anthropogenic interference.

The DIYABC analyses revealed several insights into the genetic makeup of contemporary trout populations in relation to increased historical mining activity. Firstly, our analyses revealed that the split between Hayle trout populations upstream and downstream of the highly contaminated Godolphin region of the river occurred approximately 155 years ago, c.1860. The peak exploitation of mines in the Godolphin region was from 1815 to 1850 (Atkinson 1994). Analysis of contemporary environmental data demonstrates a marked increase in copper and zinc at Godolphin (Fig. S3), and a study of the invertebrate communities within the River Hayle showed a marked decrease in species diversity in this region (Brown 1977). Thus, the present genetic structure appears indicative of rapid changes associated with significantly increased metal contaminants within the river during the Industrial Revolution. Moreover, the DIYABC results corroborate the results of population bottleneck analyses, demonstrating that Hayle trout have experienced severe population declines associated with this period of mining activity.

The second identified split (t2, Table2) demonstrated that all trout populations from metal-contaminated rivers were derived from a single common ancestor approximately 960 years ago, c.1050—during the Medieval period from whence documentary evidence of tin mining in the region first dates (Lewis 1965). In particular, this coincides with an increased demand for metals in England, due to considerable population growth (Schofield and Vince 2003), and advancements in mining technology (Hatcher 1973). The mining method of this period was predominantly tin streaming (Gerrard 2000), a process that used considerable amounts of water (Lewis 1965). For example, John de Treeures complained of the tin miners of Cornwall, ‘by reason of the great quantity of water they deluge the land where they work’ (c. 1300s); complaints of this sort were numerous throughout the Medieval period, due to the wholesale trenching and excavating for alluvial deposits in the soil (Lewis 1965).

Tin streaming had a huge impact on environmental geochemistry (Pirrie et al. 2002). Due to the low solubility of tin, the net result would not have been a significant increase in the amount of tin in the system (Weber 1985), but a likely release of other metal contaminants by liberating them from the lode (Yim 1981). A further effect was that tin mining caused huge amounts of sediment to be remobilized and carried further downstream (Thorndycraft et al. 2004), which would have had further significant environmental impacts. Salmonids are particularly sensitive to suspended solids, as they affect gravel permeability and oxygen supply in embryos (Schindler Wildhaber et al. 2014), swimming performance and physiology (Berli et al. 2014), and predator avoidance (Louhi et al. 2011). The demonstration of population bottlenecks in the DIYABC suggests that this period had detrimental effects on the populations affected by these mining practices.

It is therefore likely that the mining activity of the Medieval period, through both increased metal contamination and the sediment effects of tin streaming, would have driven changes in trout population connectivity and in-river genetic structure. Later mining activity during the Industrial Revolution would have added additional population-level pressures on trout populations already shaped in part by the effects of Medieval mining practices resulting in the genetic patterns we observe here.

We found that metal-impacted populations have reduced genetic diversity compared to relatively unaffected control populations from clean rivers. Trout from the River Hayle and Red River have very low levels of genetic variation compared to populations with little metal contamination. An exception to this pattern is the higher level of genetic diversity observed in the Trevaylor 1 population. This may be attributed to other factors: a patchwork of metal contamination (Webb et al. 1978); a negative association between the level of genetic diversity and distance from the river mouth (Primmer et al. 2006); effects of asymmetric gene flow and metapopulation dynamics (Palstra et al. 2007); or genetic instability caused by temporal fluctuations of allele frequencies within the river (Jensen et al. 2005).

These low levels of genetic diversity are likely to be one of the most commonly observed effects of exposure to environmental contaminants (Bickham et al. 2000; Van Straalen and Timmermans 2002) with several studies demonstrating evidence of dramatic genetic reductions related to metal contamination (Bourret et al. 2008; Ungherese et al. 2010; Mussali-Galante et al. 2013). Such diversity-reducing events are likely the result of population bottlenecks, which involve a temporary reduction of population size and subsequently an increase in the rate of genetic drift. The program BOTTLENECK has been shown to more accurately detect recent signatures of declines in effective population size (Beebee and Rowe 2001), whereas the recovery time of the *M*-ratio suggests that this test is more indicative of historical reductions (Garza and Williamson 2001) that persisted for a comparatively longer duration (Williamson-Natesan 2005).

Tests using BOTTLENECK showed that recent demographic declines have occurred in several of the metal-impacted populations. This is a clear genetic signature of how metal contamination may have caused local extinctions in the trout occupying these contaminated rivers. It should also be noted that two comparably clean-river populations also showed evidence of a bottleneck using this method, although the cause of these declines cannot be determined.

Low *M*-ratio values for all populations in this study, based on comparisons to the critical *M* value calculated here (0.87), an *M*-ratio of 0.76 calculated i n other studies of salmonids (Dillane et al. 2008; Frazer and Russello 2013) and the critical *M* value of 0.68 suggested by Garza and Williamson (2001) suggest that all populations have suffered population declines. Geologically, much of the southwest of Britain is dominated by metal-bearing rocks (Dines 1956; Webb et al. 1978), and with a known mining history dating back to the Bronze Age, it is possible that historical mining activity may have caused population declines in these ‘clean’ rivers in the past. In giant Amazonian river turtles (*Podocnemis expansa),* tests for demographic declines using BOTTLENECK showed that about half of populations had declined, whereas *M*-ratio values suggested all populations had suffered long-term declines as a result of hunting pressure (Pearse et al. 2006).

Our population structure analyses showed strong evidence of genetic differentiation between clean populations and those affected by metal contamination. Both population structure analyses suggest that although the ‘clean’ populations are geographically distant, they cluster together and thus constitute a relatively homogeneous group. By comparison, the metal-impacted rivers are geographically proximate, yet the genetic differentiation between the various populations places them in distinct population groups.

To our knowledge, there are no physical barriers to fish movement within the rivers studied and tests for isolation by distance were nonsignificant (Fig. S4). Therefore, the generation of genetically distinct metal groups in this study is likely the consequence of population bottlenecks and extensive genetic drift within rivers and also, a possible disruption of gene flow between rivers due to local adaptation to metal contamination. Hayle river water has been shown to be toxic to metal-naïve fish (Durrant et al. 2011). Furthermore, considerable metal accumulation in Hayle trout tissues, as well as identification of upregulated pathways involved in metal detoxification and ion homeostasis, suggests adaptation may play a role (Uren-Webster et al. 2013). The genetic patterns may suggest that the complex of different metals and their varying contamination levels within the impacted rivers are driving different underlying genetic responses. Populations of yellow perch (*Perca flavescens*) from two major mining regions in Canada showed distinct population structuring and reduced genetic variation that was correlated with cadmium concentration, but no relationship was found with levels of copper contamination (Bourret et al. 2008).

We found no significant indication that any of the loci utilized here show consistent evidence for selection. However, the striking patterns of neutral genetic divergence may suggest genetic changes elsewhere in the genome may have occurred. For example, the ecotoxicological impact of the metals may have caused large areas of the genome (‘genomic islands’) to undergo selective sweeps (Nosil and Feder 2012), and the power of just a handful of genetic markers may not be sufficient to uncover this signal. In studies looking for footprints of selection in the freshwater adaptation of three-spine stickleback (*Gasterosteus aculeatus*), only 2.8% of loci were found to be under directional selection in Baltic populations (Mäkinen et al. 2008), while nine genomic regions with significantly differentiated *F*_ST_, accounting for only ∽2.5% of the entire genome, were found in Alaskan populations (Hohenlohe et al. 2010). An applied genomics approach might help to elucidate the genomewide effects of metal contamination.

The River Hayle provided an ideal system for identifying the effects of metal contamination on trout at a local scale. We revisit previous findings, which concluded that although Hayle water had a negative effect on metal-naïve fish, there were no reductions in genetic diversity and within-river variation was not structured on the basis of metal contamination (Durrant et al. 2011). Our genetic diversity calculations showed that Hayle trout have significantly reduced variation compared to all other populations studied (both metal-impacted and clean). Interestingly, genetic diversity was higher at the upstream sites, the opposite of what we might expect given that gene flow in salmonids tends to be downstream, therefore increasing genetic diversity in downstream sites (Griffiths et al. 2009). These unusual patterns of genetic diversity and structure may be due to the effects of Godolphin mine washout and any waterborne contaminants are likely to be moving with the flow of the river, enforcing higher ecotoxicological pressures on populations downstream of the contaminated region. Metal contamination is considerably higher downstream of Godolphin (total zinc ∽570 μg/L; total copper 36 μg/L; total arsenic ∽11 μg/L) compared to the upstream section of the catchment (total zinc ∽36 μg/L; total copper ∽6 μg/L; total arsenic ∽3 μg/L).

Our analyses of the within-genetic structuring of the Hayle reveal that the upper and lower regions are highly spatially structured, despite being just 7.7 km apart. While olfaction is a critically important behavior in salmonids and is known to be negatively affected by metal ions (Hansen et al. 1999; Baldwin et al. 2003), in this instance, the strength of the barrier presented by the highly metal-impacted Godolphin zone appears to act to maintain structure in spite of any possible reduction in fidelity (Tierney et al. 2010) of homing by returning adult trout caused by increased metal ion levels in the river water. Metal concentrations in the Godolphin section of the Hayle are extremely high (total zinc ∽2512 μg/L; total copper ∽471 μg/L; total arsenic ∽99 μg/L) and are thus likely to be restricting gene flow and reinforcing in-river population structure, through a combination of avoidance behavior (Woodward et al. 1995) and direct mortality on fish attempting to traverse the Godolphin toxic zone. Thus, the impact of this chemical barrier appears to restrict fish movement to the same extent as physical barriers observed in some other systems (Hansen et al. 2014).

## Conclusions

Evolutionary change can be associated with human activities, and such disturbance history has been suggested to be influential on the genetic architecture of natural populations (Stockwell et al. 2003; Banks et al. 2013). We have shown that mining for metals in the southwest region of Britain has resulted in striking patterns of genetic diversity and population structure both within and between river systems. These changes have arisen both during an early period of increased mining intensity—the Medieval period —and later during the Industrial Revolution. We demonstrate evidence that the observed neutral genetic divergence is due to population bottlenecks, disruptions of gene flow, and a likely increased rate of genetic drift. However, there is also a possibility that metal-impacted trout have developed a genetic adaptation—this cannot be explored here, but future studies should develop on understanding the broader genomic effects, as well as the physiology and molecular mechanisms of potential metal tolerance.

## Data archiving statement

Data available from the Dryad Digital Repository: http://dx.doi.org/10.5061/dryad.j3127

Microsatellite genotypes are available in Dryad (doi: 10.5061/dryad.j3127).

Data on metal contaminant levels are available through the Environment Agency South West Enquiries [SouthWestEnquiries@environment-agency.gov.uk].

## Supporting Information

**Figure S1.** DIYABC scenarios tested on the various population groups.

**Figure S2.** Tests for loci under selection using Bayescan (A), *lnRV* (B) and Fdist (C).

**Figure S3.** from the Environment Agency (1990–2014) on concentrations of metals (µg/L) at the sites across the River Hayle.

**Figure S4.** Relationship between genetic distance (FST/(1-FST)) and geographic distance (kilometres) for the 15 populations of brown trout, based on 99 permutations; rxy = −0.09, *P* = 0.28, *R*^2^ = 0.0082.

**Table S1.** Location and site identification for each sampled population.

**Table S2.** Details of the eight multiplexes including quantities of forward and reverse primer, total volumes and PCR touchdown (TD) programme.

**Table S3.** Pairwise *F*_ST_ results for each of the 15 brown trout populations.
