# Supplementary material for: Human mining activity across the ages determines the genetic structure of modern brown trout (Salmo trutta L.) populations
Source: Evol Appl. 2015 May 28;8(6):573–85. doi: 10.1111/eva.12266 (PMC4479513; doi:10.1111/eva.12266)
Supplement: Supplementary file 1 [file eva0008-0573-sd1.docx]

**Supporting Information: 5.** DIYABC scenarios tested on the various population groups. Group 1: populations are derived multiple times from a common ancestor. Group 2: metal populations are derived multiple times from a general clean lineage. Group 3: the clean and metal groups are separate lineages, with populations from metal contaminated sites being derived from the common metal-contaminated lineage. Scenarios surrounded by a red box were shown to be the most likely, based on logistic regression of the posterior probabilities of 10^5^ simulations per scenario. These four scenarios were compared against one another in further simulations (10^6^ simulations per scenario).

**
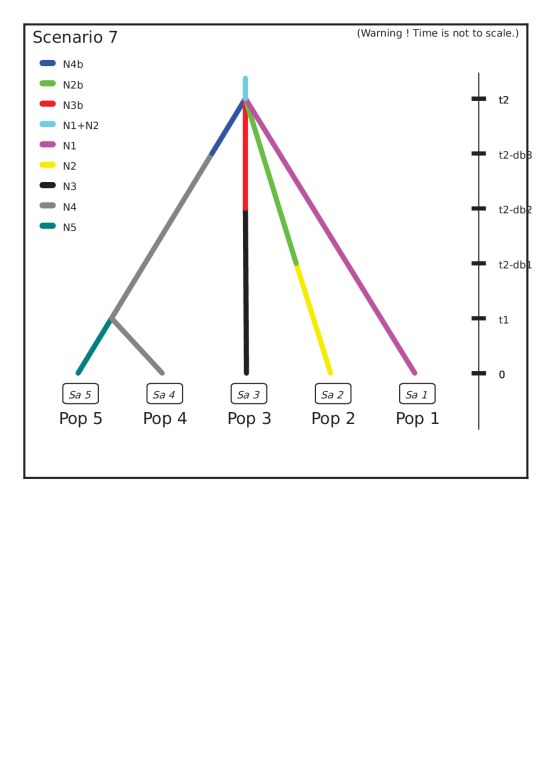

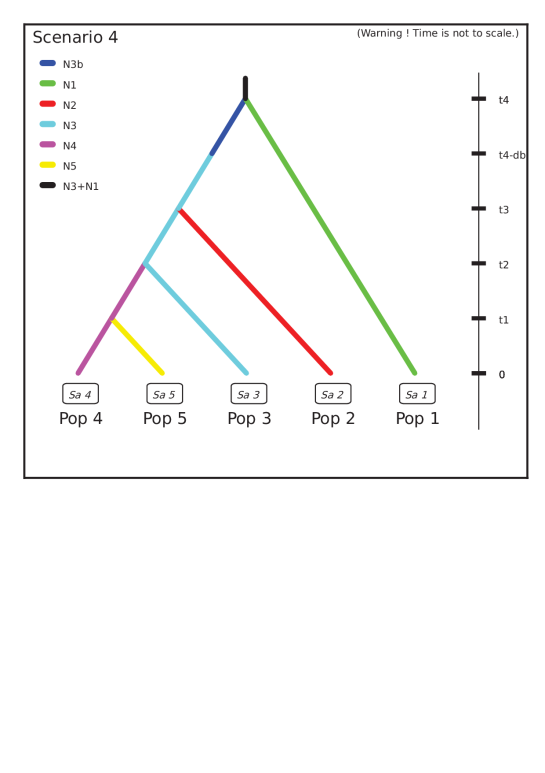

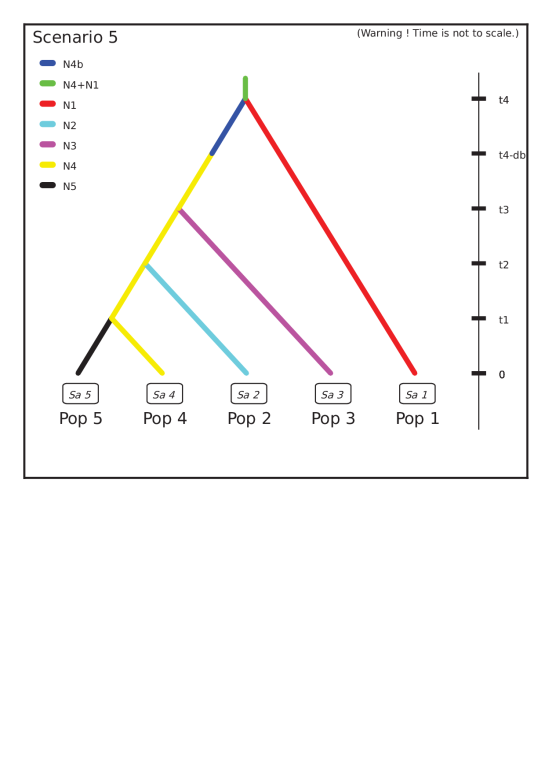

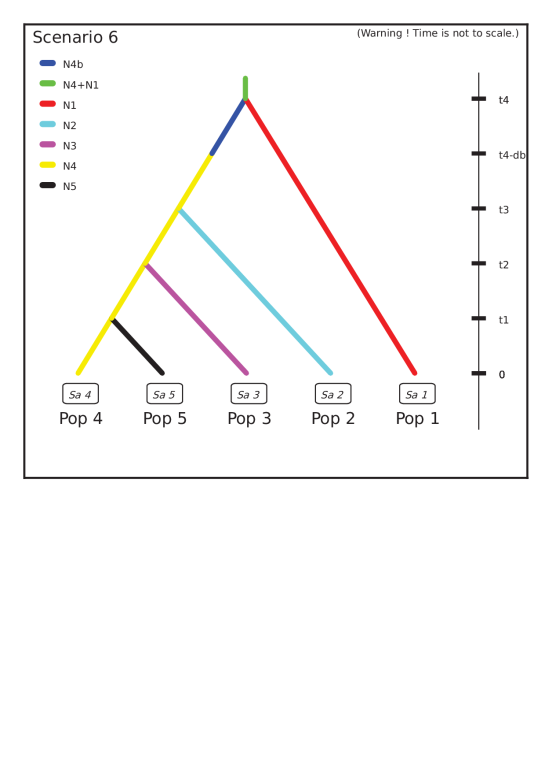

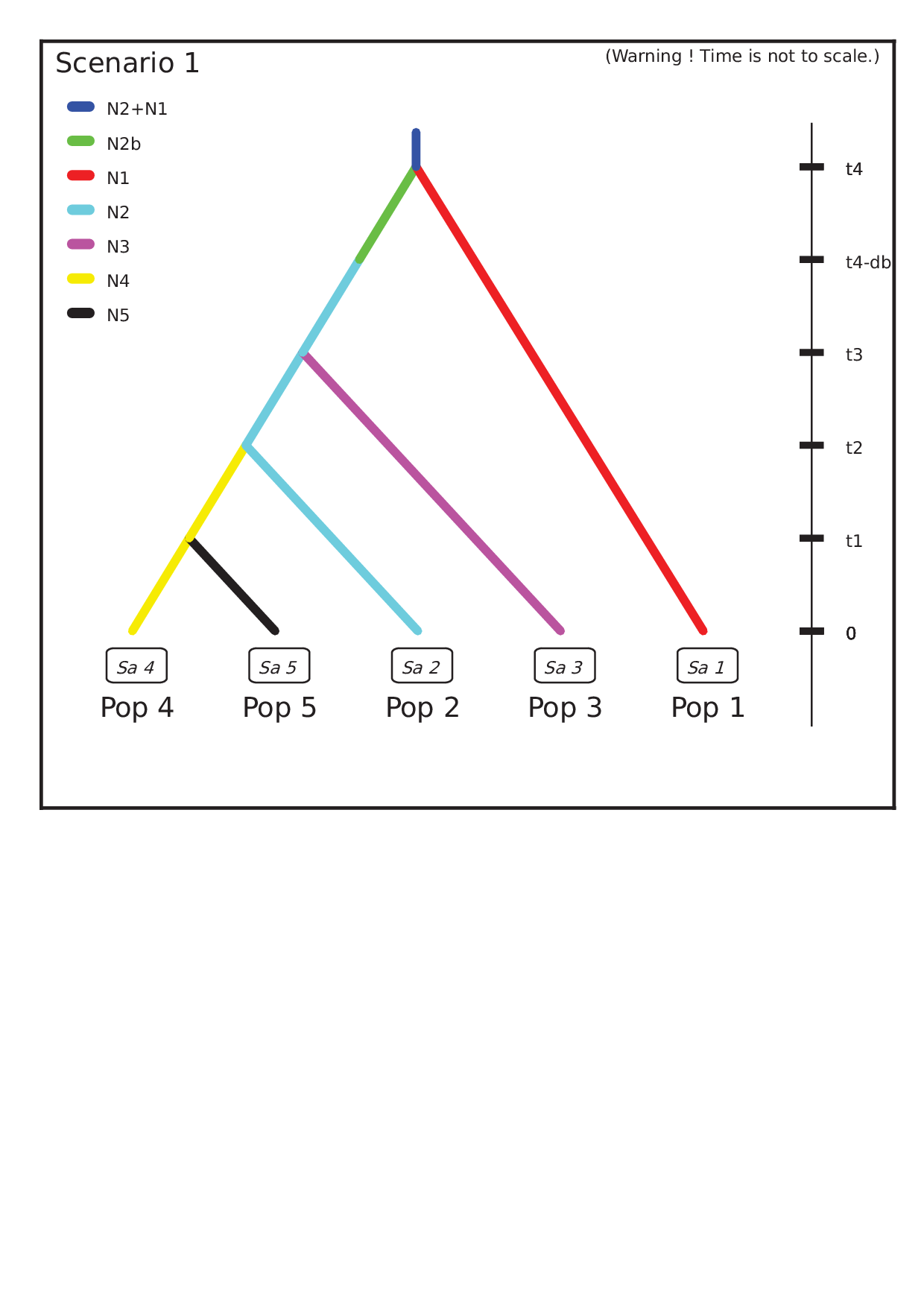

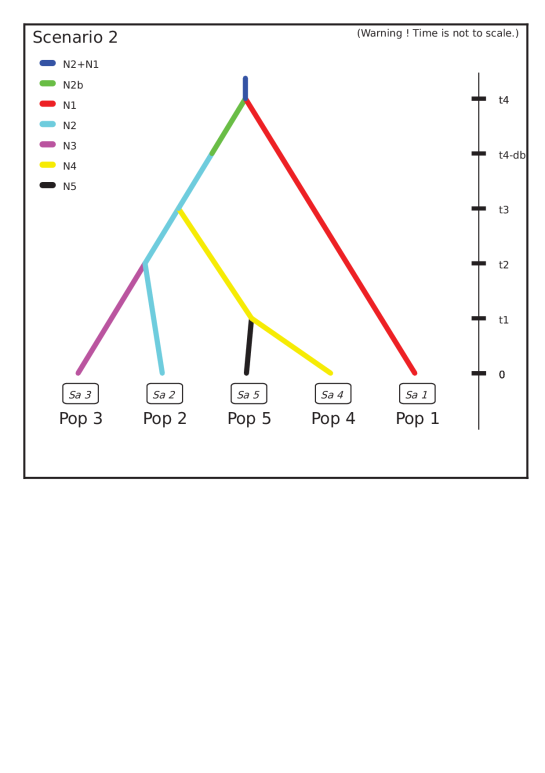

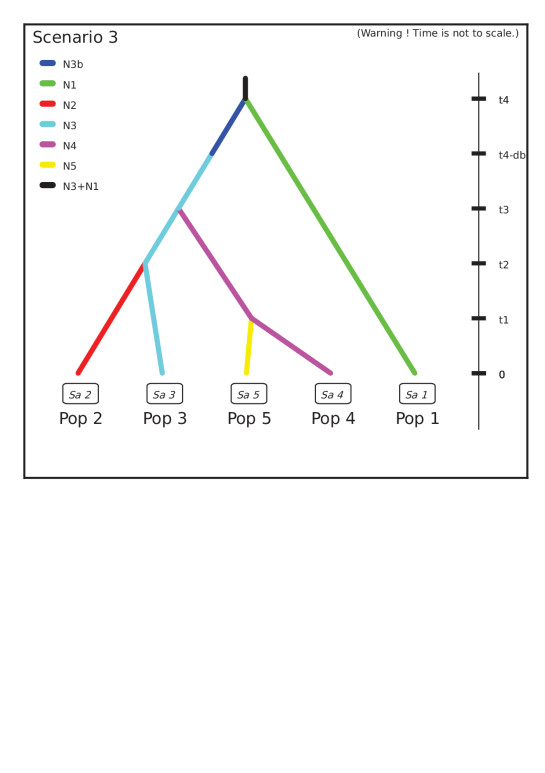
**

**Group 1**

**Common ancestor scenarios**


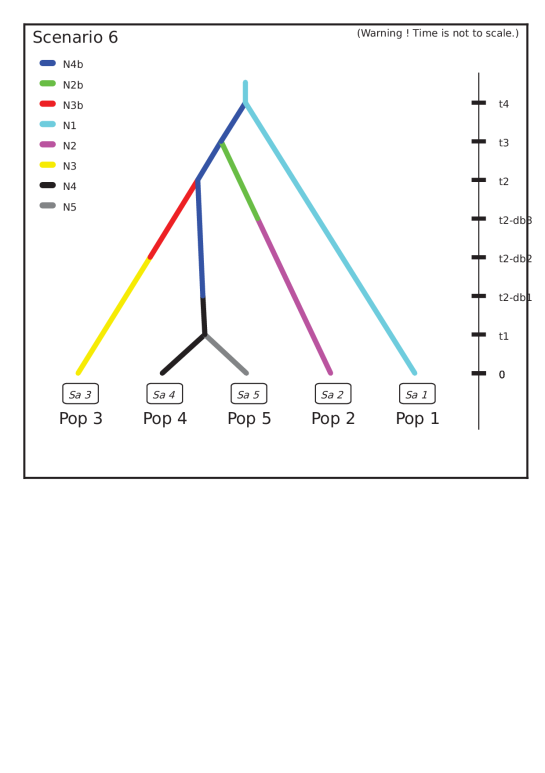

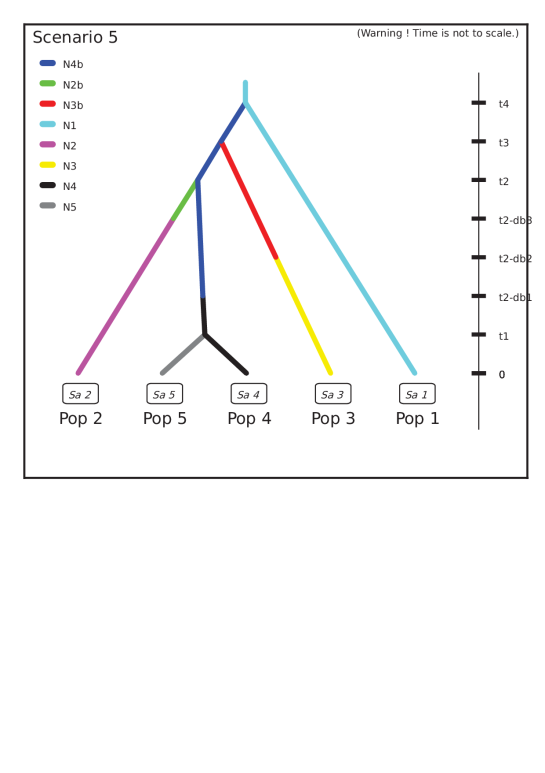

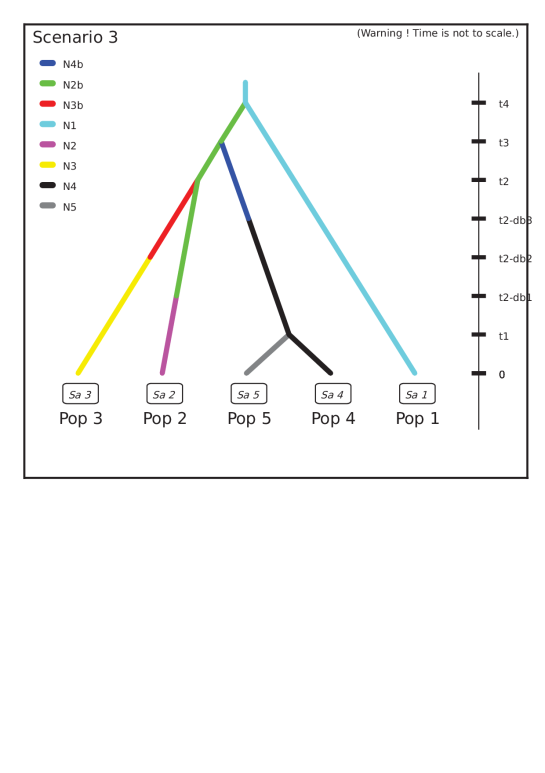

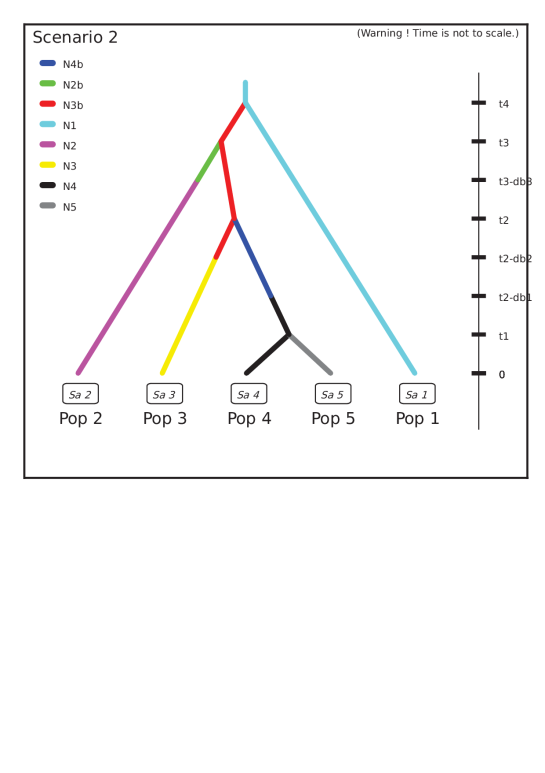

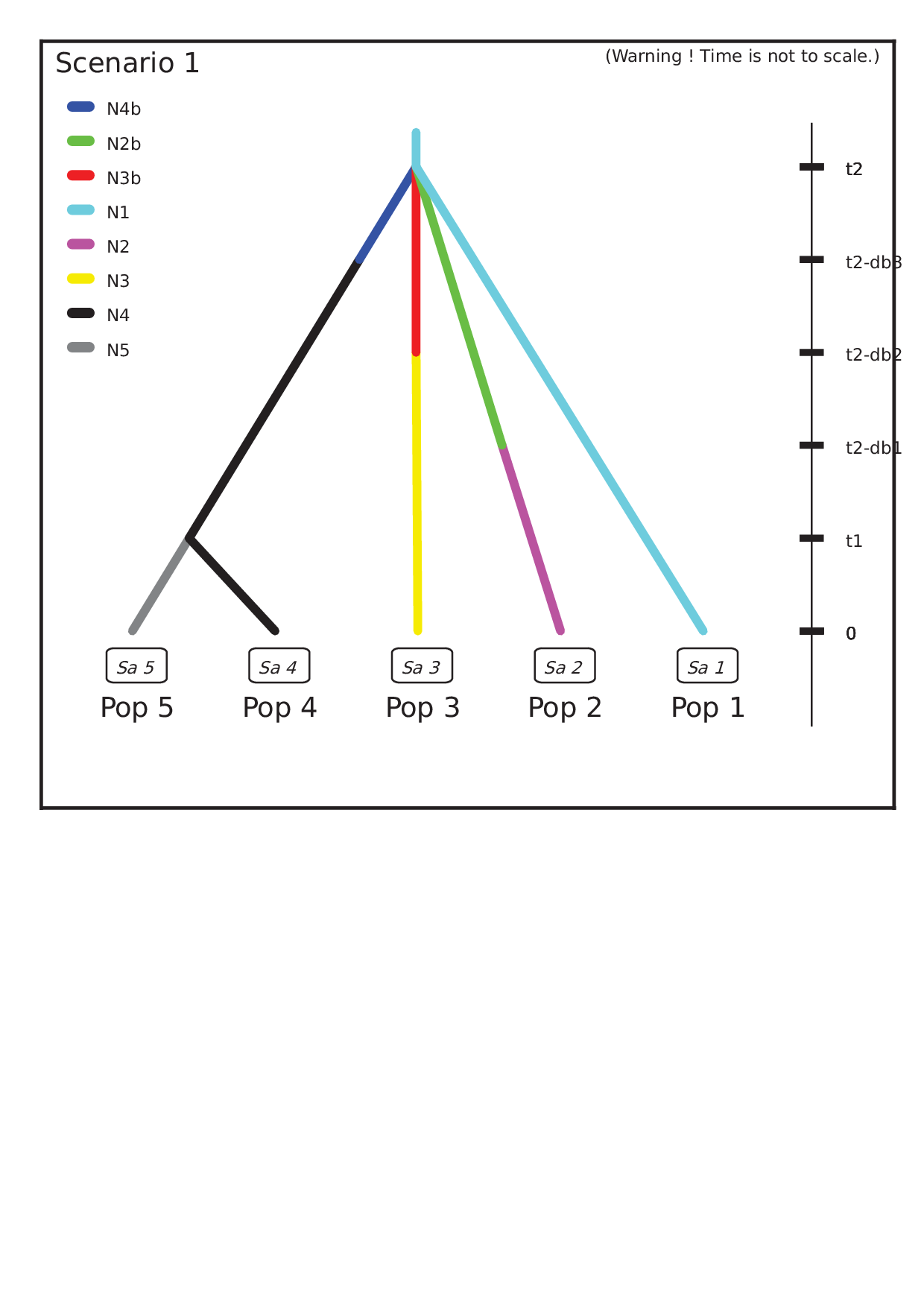

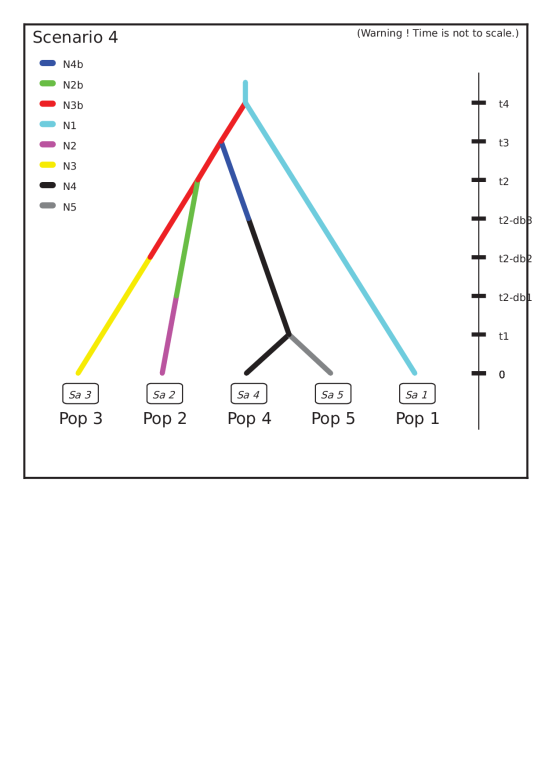


**Group 2**

**Clean lineage scenarios**


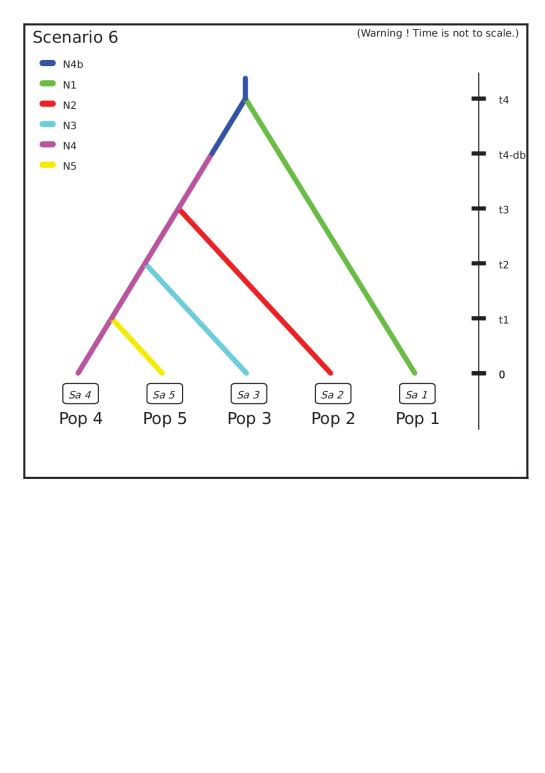

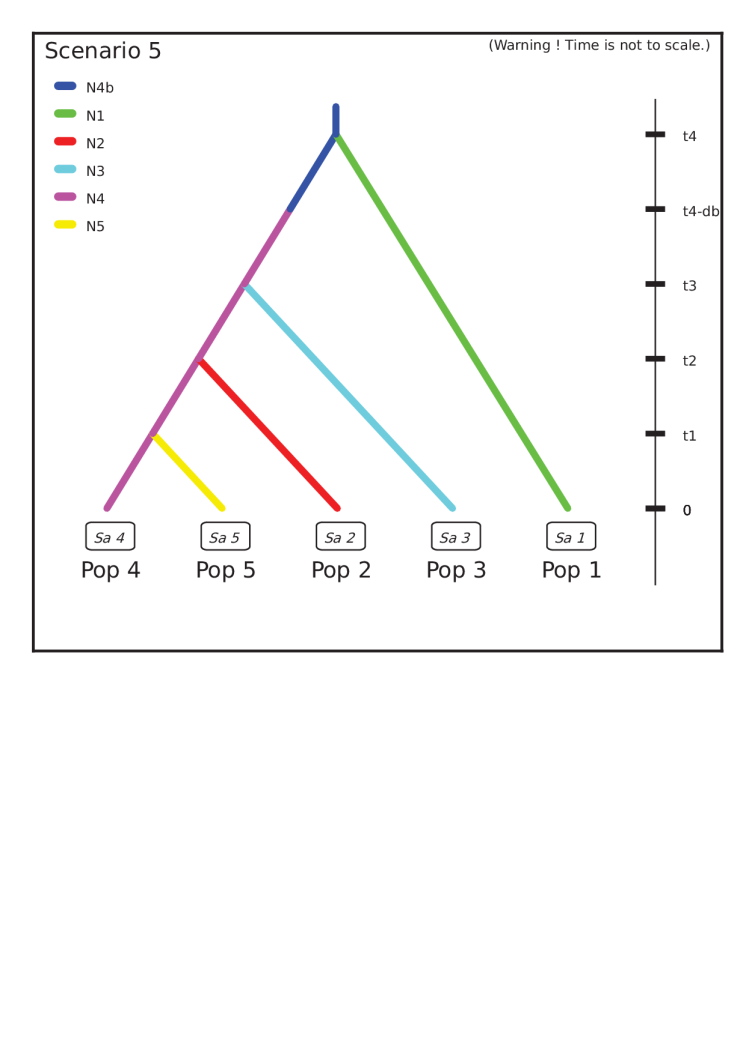

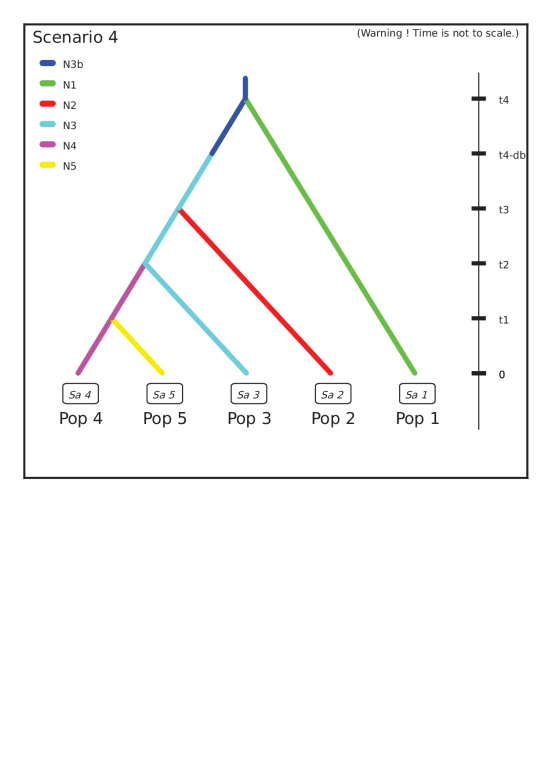

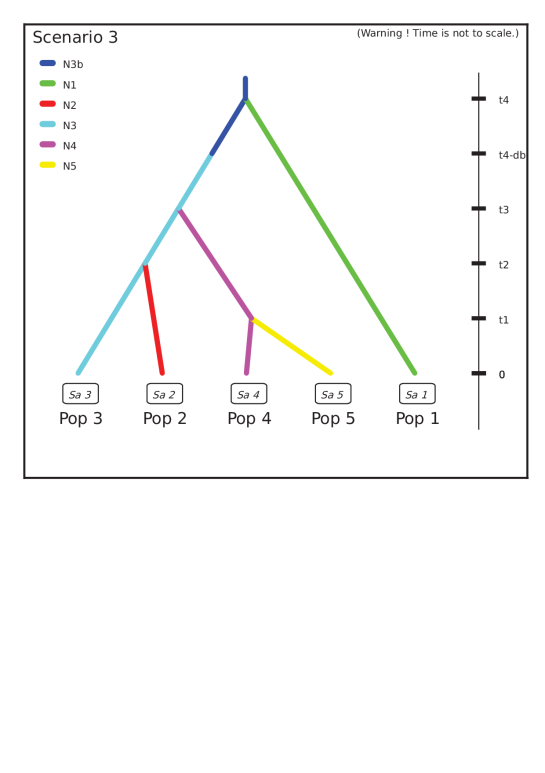

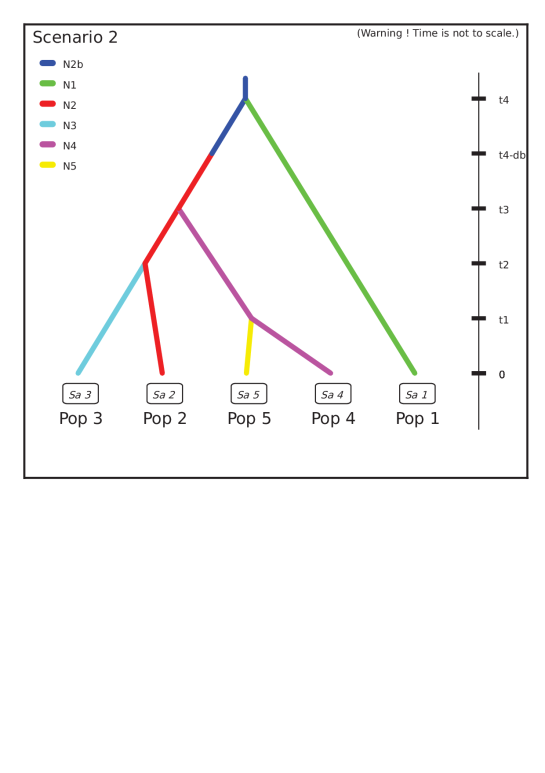

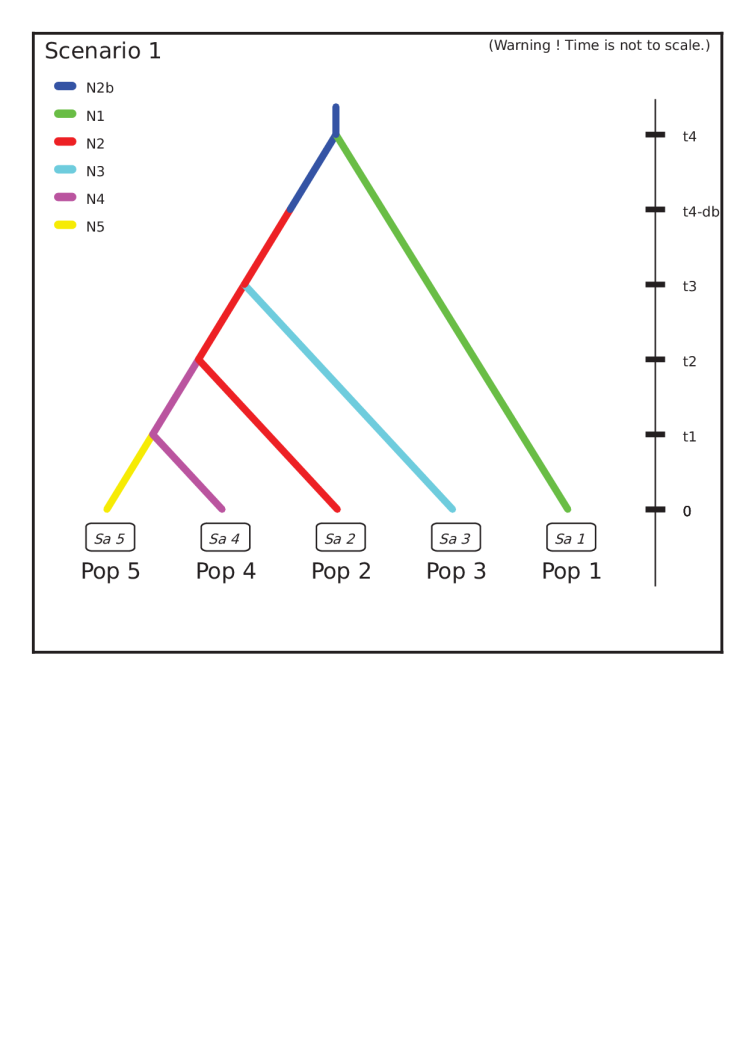


**Group 3**

**Metal lineage scenarios**
