# Supplementary material for: Human mining activity across the ages determines the genetic structure of modern brown trout (Salmo trutta L.) populations
Source: Evol Appl. 2015 May 28;8(6):573–85. doi: 10.1111/eva.12266 (PMC4479513; doi:10.1111/eva.12266)
Supplement: Supplementary file 2 [file eva0008-0573-sd2.docx]

**Supporting Information: Figure 2.** Tests for loci under selection using Bayescan (**2A**), *lnRV* (**2B**) and Fdist (**2C**)

SsaD157

Ssa407UOS

One102b

Ssa407UOS

SsaD157

Ssa52NVH

SsaD58

**(i)**

**(ii)**


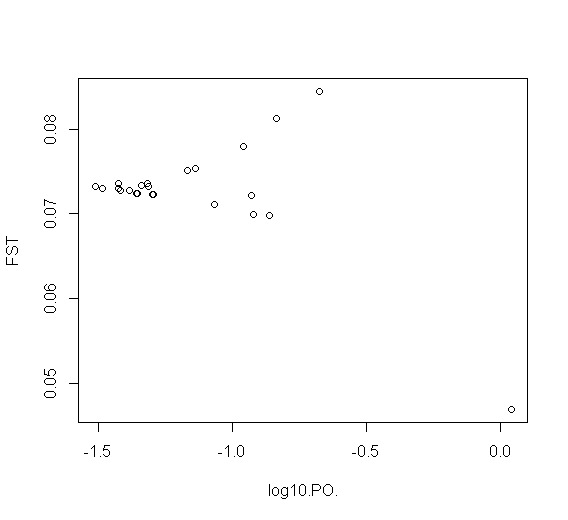

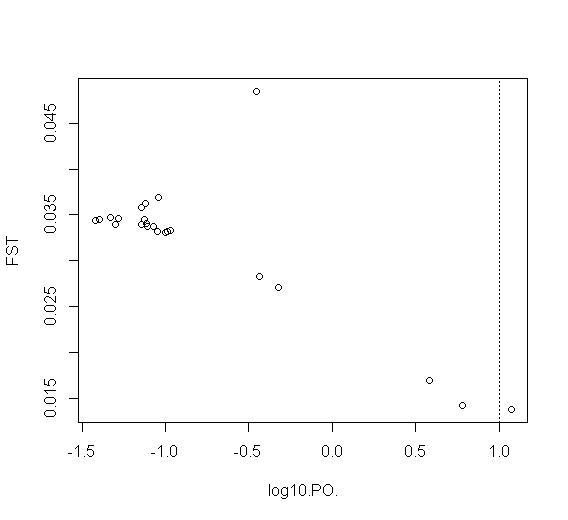

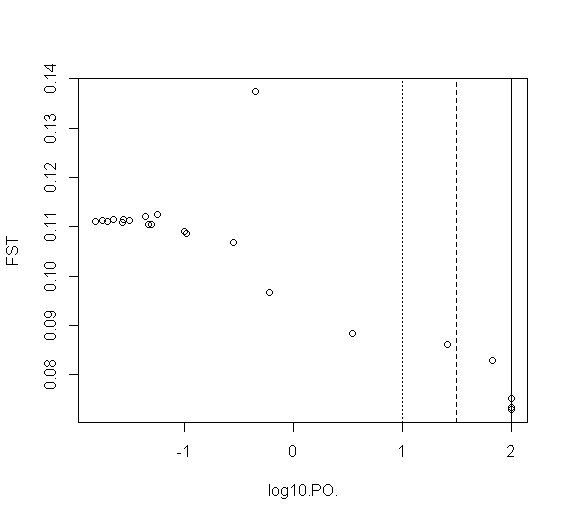

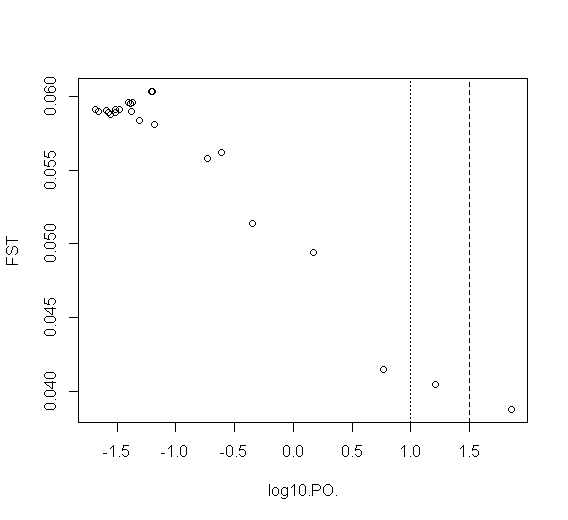
**Supplementary Information: Figure 2A.** Bayescan results using FDIST method. Log10 Posterior Odds (PO) value against F_ST_. Vertical lines represent cut-off values for posterior odds: dotted line >1; dashed line >1.5; solid line > 2. Some loci analysed have a Log10 Posterior Odds value of 1000; these are represented on the graph as 2. (i) All populations. (ii) Clean populations. (iii) Hayle populations. (iv) Crowlas and Trevaylor populations.

SsaD58

**(iv)**

**(iii)**


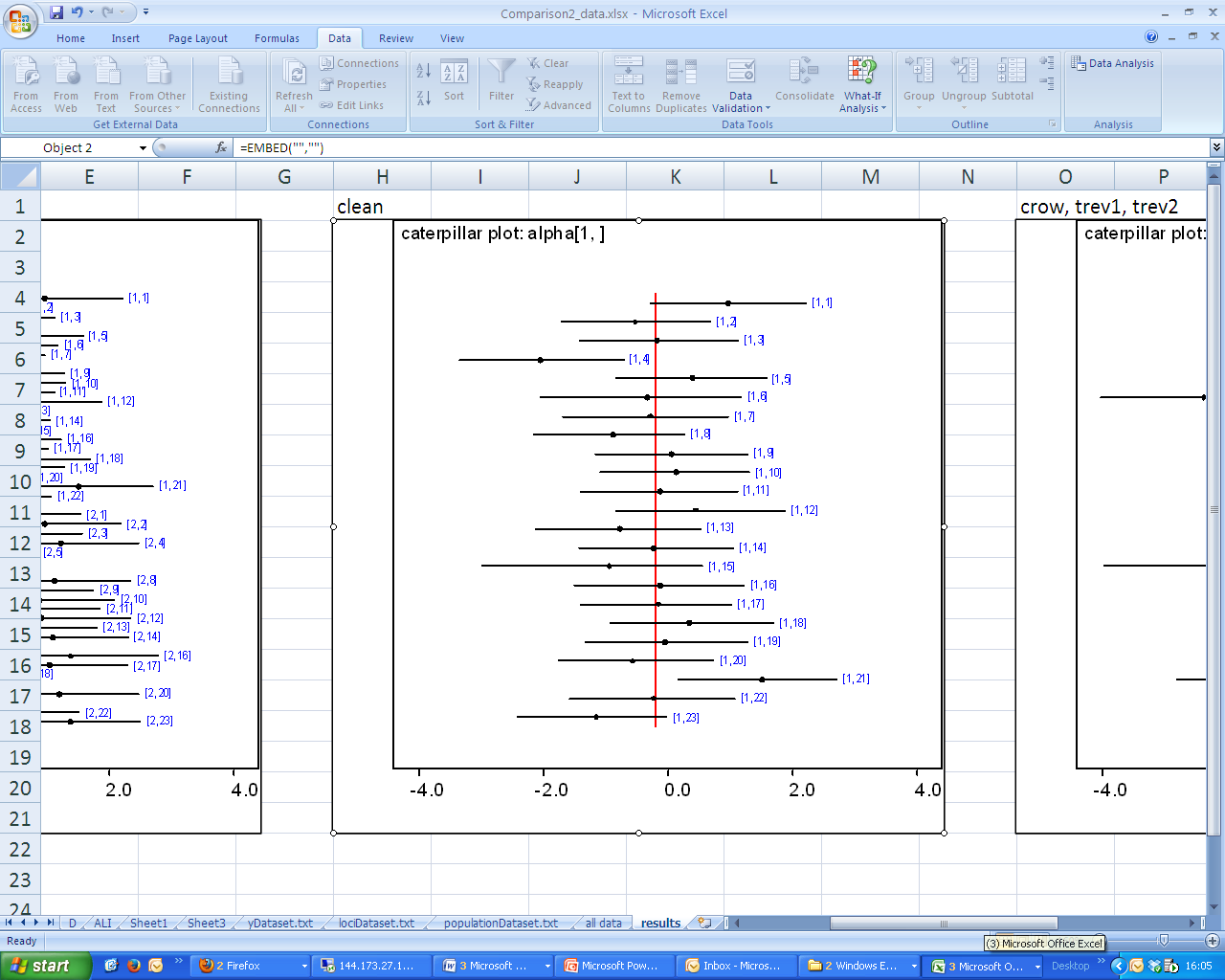

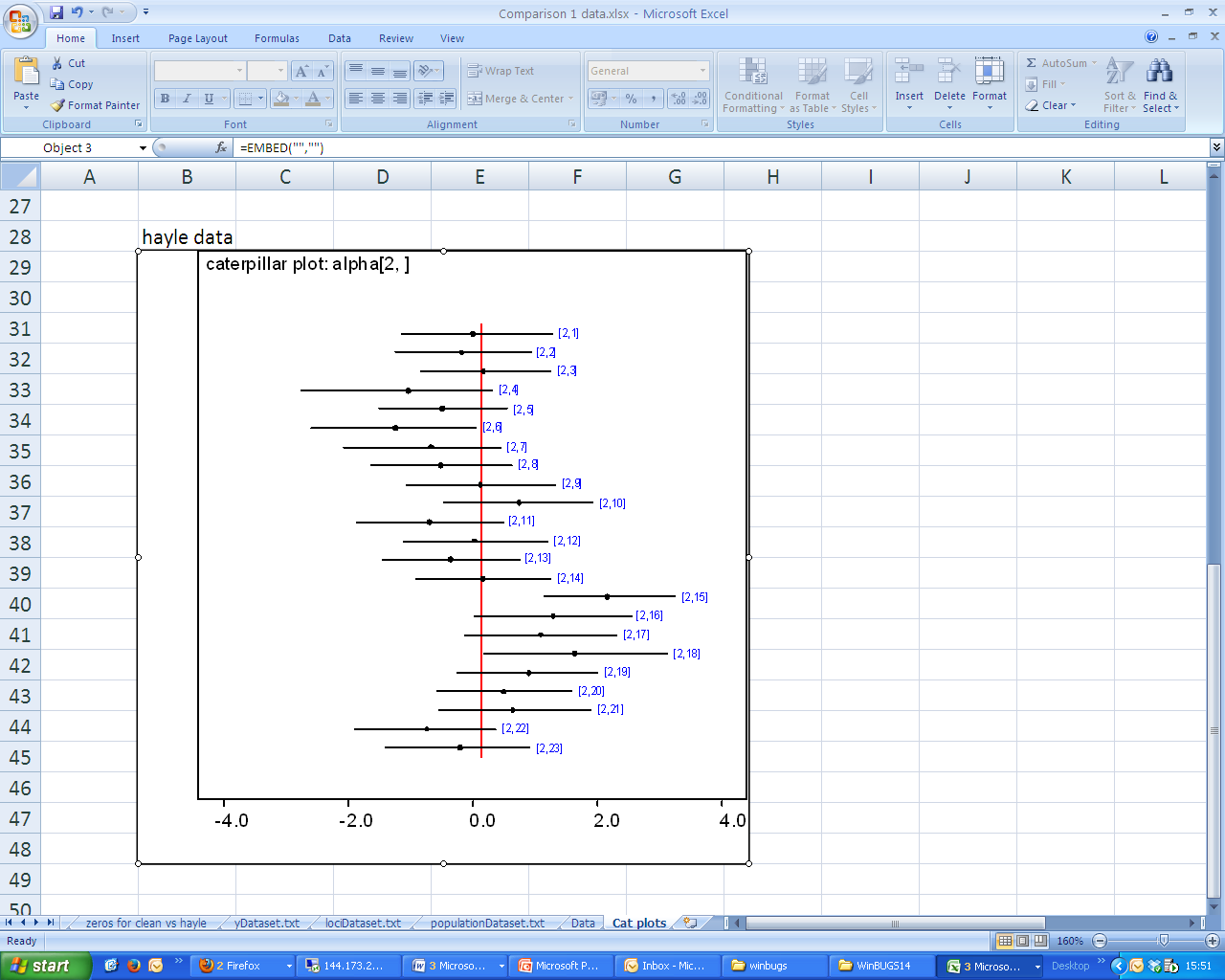

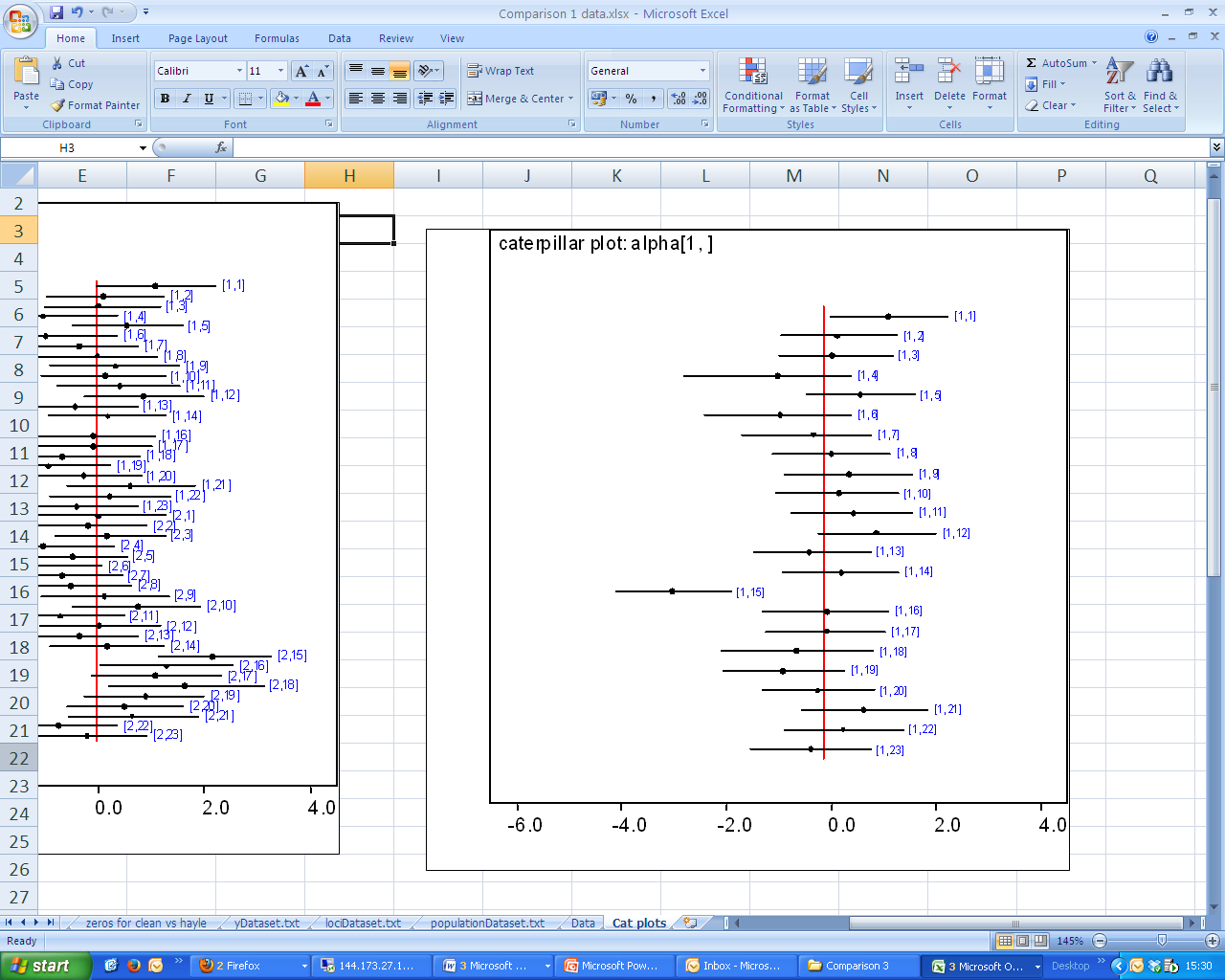

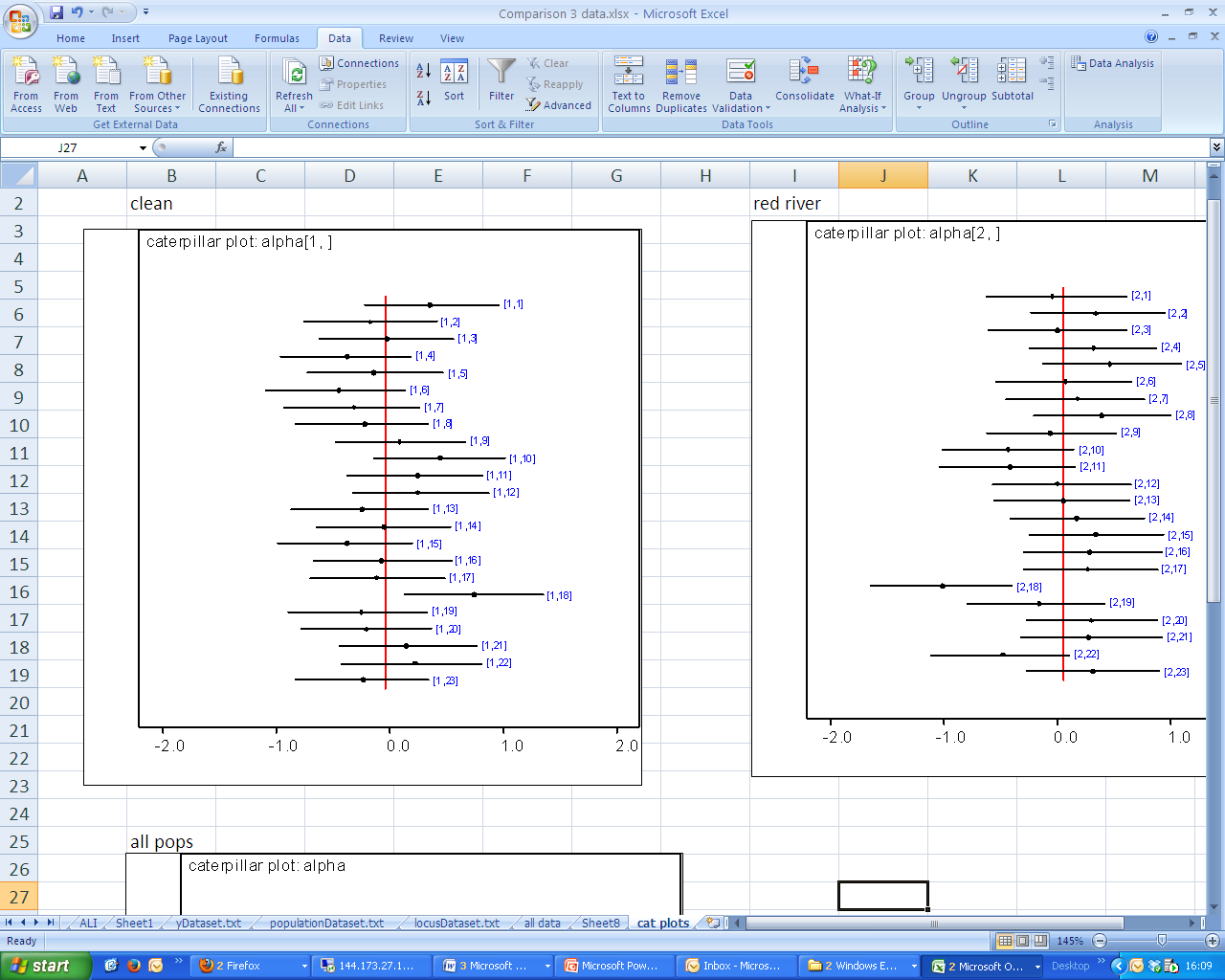

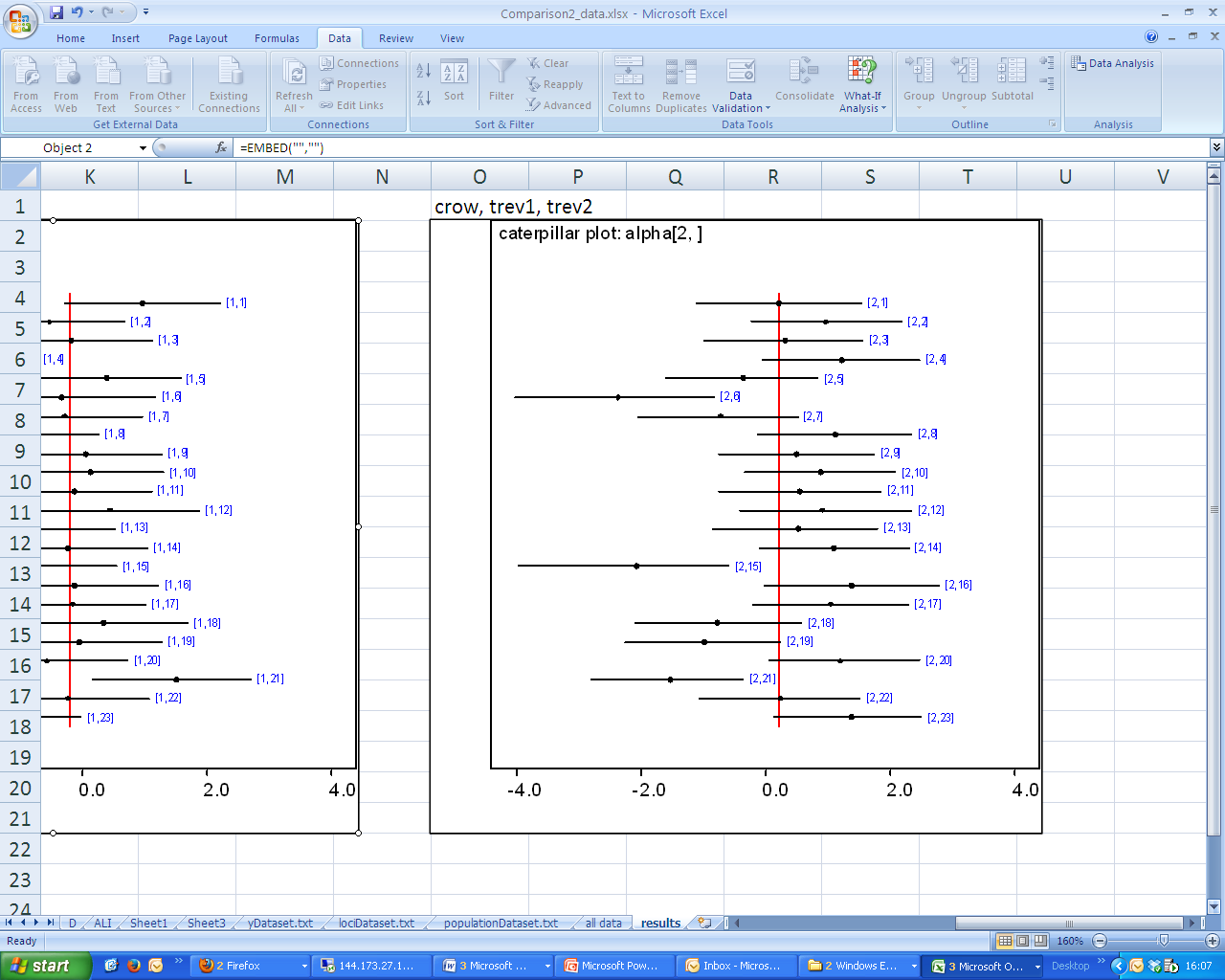

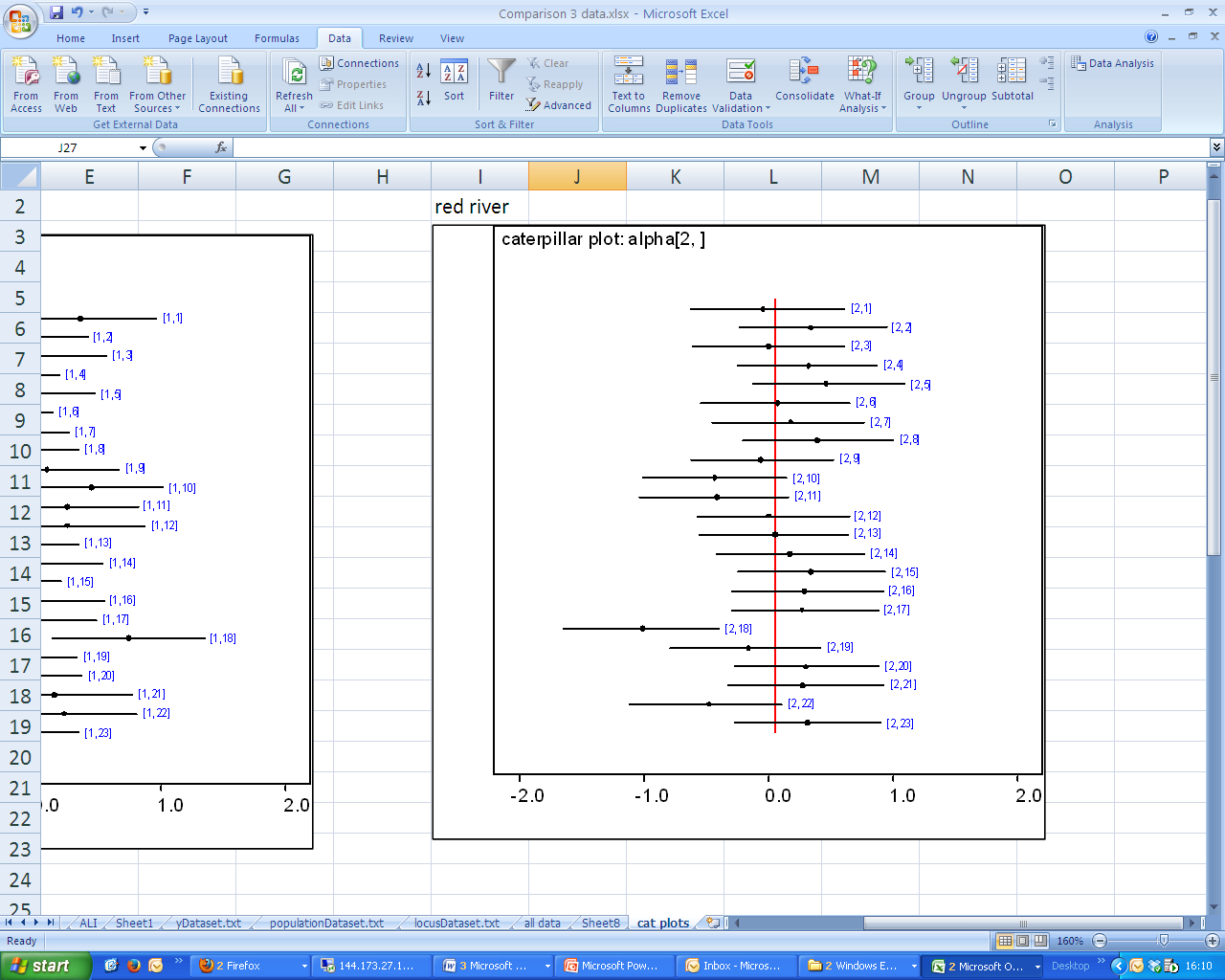


**(i)**

**(ii)**

**(iii)**

**Supplementary Information: Figure 2B.** Caterpillar plots showing posterior distributions of α_ij_-values used to detect selective sweeps using the *lnRV* statistic. Median values of α_ij_ > 0.5 are indicative of selection. We considered α_ij_-values >1 to be indicative of strong selection. (i) Comparison 1 – Clean vs. Hayle populations. Loci with high α_ij_-values: One102a & Ssa85. (ii) Comparison 2 – Clean vs. CROW/TREV1/TREV2. Loci with high α_ij_-values: Ssa85, CA060208, One102a, Ssa412 & TAP2B. (iii) Comparison 3 – Clean vs. Red River populations. Loci with high α_ij_-values: sasaTAP2A. Caterpillar plot bounds represent lower (2.5%) and upper (97.5%) quartiles of the Bayesian interval.

**Supplementary Information: Figure 2C.** Table of results from the Fdist test, implemented in Arlequin,. (i) Comparison 1 - Clean vs. Hayle populations. (ii) Comparison 2 – Clean vs. CROW/TREV1/TREV2. (iii) Comparison 3 – Clean vs. Red River populations. (iv) Comparison 4 – Clean vs. all metal populations. p values <0.01 (in red) are significant.

**(i)**

| **Locus** | **HO** | **FCT** | **P-value run 1** | **P-value run 2** | **P-value run 3** |
| --- | --- | --- | --- | --- | --- |
| Ssa52NVH | 1.0031 | 0.1328 | 0.0094 | 0.0117 | 0.0106 |
| CA048828 | 0.9631 | 0.0727 | 0.1638 | 0.1700 | 0.1720 |
| BG935488 | 0.7620 | 0.0314 | 0.1823 | 0.1826 | 0.1835 |
| CA060208 | 0.6338 | 0.0661 | 0.4513 | 0.4570 | 0.4351 |
| SS11 | 0.8578 | 0.1249 | 0.2056 | 0.1949 | 0.2011 |
| Ssa85 | 0.6412 | 0.1615 | 0.1437 | 0.1488 | 0.1399 |
| SsosL417 | 0.8863 | 0.0363 | 0.1736 | 0.1832 | 0.1715 |
| CA060177 | 0.8627 | 0.0344 | 0.1572 | 0.1614 | 0.1491 |
| SsosL311 | 0.9498 | 0.0589 | 0.3400 | 0.3483 | 0.3485 |
| Ssa407UOS | 0.9708 | 0.0557 | 0.2310 | 0.2324 | 0.2412 |
| Str3QUB | 0.5164 | 0.0398 | 0.3402 | 0.3355 | 0.3252 |
| SsaD58 | 0.9743 | 0.0525 | 0.2385 | 0.2383 | 0.2476 |
| SsaF43 | 0.8127 | 0.1280 | 0.2099 | 0.2273 | 0.2199 |
| SsaD157 | 0.9776 | 0.0666 | 0.1380 | 0.1403 | 0.1450 |
| One102a | 0.4853 | 0.2349 | 0.0400 | 0.0430 | 0.0492 |
| One102b | 0.9325 | 0.0321 | 0.2086 | 0.2153 | 0.2063 |
| SSsp2213 | 0.9223 | 0.0749 | 0.3756 | 0.3686 | 0.3687 |
| sasaTAP2A | 0.8471 | 0.1297 | 0.1937 | 0.1897 | 0.1930 |
| Ssa197 | 0.8906 | 0.1763 | 0.0614 | 0.0556 | 0.0577 |
| CA515794 | 0.7988 | 0.0584 | 0.3837 | 0.3712 | 0.3781 |
| Ssa412UOS | 0.7939 | 0.2163 | 0.0513 | 0.0656 | 0.0593 |
| CA769358 | 0.8262 | 0.0551 | 0.3551 | 0.3414 | 0.3328 |
| sasaTAP2B | 0.6014 | 0.0827 | 0.3738 | 0.3747 | 0.3666 |

**(ii)**

| **Locus** | **HO** | **FCT** | **P-value run 1** | **P-value run 2** | **P-value run 3** |
| --- | --- | --- | --- | --- | --- |
| Ssa52NVH | 0.9677 | 0.0219 | 0.4227 | 0.4189 | 0.4221 |
| CA048828 | 0.9558 | 0.0583 | 0.0821 | 0.0838 | 0.0860 |
| BG935488 | 0.8212 | 0.0332 | 0.3946 | 0.3921 | 0.4017 |
| CA060208 | 0.7407 | 0.0934 | 0.1205 | 0.1150 | 0.1229 |
| SS11 | 0.9116 | 0.0497 | 0.2893 | 0.2823 | 0.2981 |
| Ssa85 | 0.5555 | 0.1960 | 0.0325 | 0.0395 | 0.0356 |
| SsosL417 | 0.8896 | 0.0249 | 0.2861 | 0.2877 | 0.2825 |
| CA060177 | 0.8893 | 0.0484 | 0.3343 | 0.3249 | 0.3387 |
| SsosL311 | 0.9305 | 0.0009 | 0.0063 | 0.0060 | 0.0059 |
| Ssa407UOS | 0.9627 | 0.0188 | 0.3397 | 0.3362 | 0.3391 |
| Str3QUB | 0.5622 | 0.0101 | 0.1217 | 0.1191 | 0.1209 |
| SsaD58 | 0.9840 | 0.0559 | 0.0366 | 0.0375 | 0.0393 |
| SsaF43 | 0.8121 | 0.0432 | 0.4122 | 0.4142 | 0.3949 |
| SsaD157 | 0.9603 | 0.0223 | 0.4075 | 0.4028 | 0.4052 |
| One102a | 0.4878 | 0.1822 | 0.0258 | 0.0256 | 0.0231 |
| One102b | 0.9331 | 0.0327 | 0.4675 | 0.4720 | 0.4645 |
| SSsp2213 | 0.9163 | 0.0336 | 0.4518 | 0.4530 | 0.4400 |
| sasaTAP2A | 0.7903 | -0.0094 | 0.0364 | 0.0349 | 0.0381 |
| Ssa197 | 0.7899 | 0.0187 | 0.1992 | 0.2067 | 0.2127 |
| CA515794 | 0.8685 | 0.0448 | 0.3842 | 0.3836 | 0.3862 |
| Ssa412UOS | 0.7993 | 0.0133 | 0.1337 | 0.1362 | 0.1427 |
| CA769358 | 0.7995 | 0.0955 | 0.1087 | 0.1059 | 0.1019 |
| sasaTAP2B | 0.5551 | 0.0195 | 0.2250 | 0.2176 | 0.2219 |

**(iii)**

| **Locus** | **HO** | **FCT** | **P-value run 1** | **P-value run 2** | **P-value run 3** |
| --- | --- | --- | --- | --- | --- |
| Ssa52NVH | 0.9800 | 0.0399 | 0.1249 | 0.1180 | 0.1193 |
| CA048828 | 0.9583 | 0.0872 | 0.0115 | 0.0131 | 0.0126 |
| BG935488 | 0.7728 | 0.0242 | 0.3114 | 0.3057 | 0.3060 |
| CA060208 | 0.6112 | 0.0148 | 0.1927 | 0.1839 | 0.1868 |
| SS11 | 0.8398 | 0.0254 | 0.3297 | 0.3372 | 0.3297 |
| Ssa85 | 0.5742 | 0.0536 | 0.2647 | 0.2789 | 0.2735 |
| SsosL417 | 0.9027 | 0.0496 | 0.2375 | 0.2483 | 0.2409 |
| CA060177 | 0.8776 | 0.0401 | 0.3647 | 0.3767 | 0.3693 |
| SsosL311 | 0.9756 | 0.0680 | 0.0212 | 0.0231 | 0.0230 |
| Ssa407UOS | 0.9743 | 0.0307 | 0.2562 | 0.2443 | 0.2483 |
| Str3QUB | 0.6356 | 0.0527 | 0.2531 | 0.2463 | 0.2612 |
| SsaD58 | 0.9734 | 0.0376 | 0.1642 | 0.1553 | 0.1573 |
| SsaF43 | 0.7882 | 0.0076 | 0.0848 | 0.0839 | 0.0836 |
| SsaD157 | 0.9747 | 0.0515 | 0.0640 | 0.0622 | 0.0616 |
| One102a | 0.4876 | 0.0337 | 0.3003 | 0.3010 | 0.2914 |
| One102b | 0.9217 | 0.0336 | 0.4198 | 0.4162 | 0.4181 |
| SSsp2213 | 0.9247 | 0.0595 | 0.1273 | 0.1294 | 0.1265 |
| sasaTAP2A | 0.7880 | -0.0094 | 0.0544 | 0.0512 | 0.0547 |
| Ssa197 | 0.7878 | 0.0050 | 0.0543 | 0.0537 | 0.0535 |
| CA515794 | 0.8055 | 0.0019 | 0.0187 | 0.0191 | 0.0186 |
| Ssa412UOS | 0.8217 | 0.0592 | 0.2224 | 0.2048 | 0.2137 |
| CA769358 | 0.8343 | 0.0444 | 0.3341 | 0.3213 | 0.3339 |
| sasaTAP2B | 0.5645 | 0.0184 | 0.2248 | 0.2154 | 0.2226 |

**(iv)**

| **Locus** | **HO** | **FCT** | **P-value run 1** | **P-value run 2** | **P-value run 3** |
| --- | --- | --- | --- | --- | --- |
| Ssa52NVH | 0.9602 | 0.0471 | 0.0393 | 0.0433 | 0.0407 |
| CA048828 | 0.9437 | 0.0475 | 0.0704 | 0.0721 | 0.0710 |
| BG935488 | 0.7810 | 0.0025 | 0.0339 | 0.0330 | 0.0340 |
| CA060208 | 0.6803 | 0.0398 | 0.2672 | 0.2613 | 0.2708 |
| SS11 | 0.8525 | 0.0133 | 0.2022 | 0.2011 | 0.2057 |
| Ssa85 | 0.5532 | -0.0338 | 0.0015 | 0.0017 | 0.0019 |
| SsosL417 | 0.8682 | 0.0318 | 0.3491 | 0.3453 | 0.3426 |
| CA060177 | 0.8729 | 0.0209 | 0.3462 | 0.3484 | 0.3510 |
| SsosL311 | 0.9343 | 0.0189 | 0.3579 | 0.3582 | 0.3538 |
| Ssa407UOS | 0.9604 | 0.0116 | 0.2498 | 0.2464 | 0.2465 |
| Str3QUB | 0.5392 | -0.0018 | 0.2384 | 0.2442 | 0.2395 |
| SsaD58 | 0.9649 | 0.0245 | 0.2712 | 0.2787 | 0.2769 |
| SsaF43 | 0.8014 | 0.0487 | 0.1808 | 0.1911 | 0.1878 |
| SsaD157 | 0.9612 | 0.0314 | 0.1608 | 0.1679 | 0.1660 |
| One102a | 0.4243 | 0.1921 | 0.0068 | 0.0106 | 0.0067 |
| One102b | 0.9192 | 0.0042 | 0.0553 | 0.0556 | 0.0542 |
| SSsp2213 | 0.9055 | 0.0377 | 0.2400 | 0.2353 | 0.2443 |
| sasaTAP2A | 0.8009 | 0.0244 | 0.3911 | 0.3829 | 0.3900 |
| Ssa197 | 0.8193 | 0.0308 | 0.3658 | 0.3741 | 0.3715 |
| CA515794 | 0.8235 | 0.0116 | 0.1735 | 0.1700 | 0.1726 |
| Ssa412UOS | 0.7374 | 0.0757 | 0.0773 | 0.0807 | 0.0785 |
| CA769358 | 0.7847 | 0.0106 | 0.1612 | 0.1562 | 0.1614 |
| sasaTAP2B | 0.5739 | -0.0103 | 0.1207 | 0.1206 | 0.1120 |
