# Supplementary material for: Human mining activity across the ages determines the genetic structure of modern brown trout (Salmo trutta L.) populations
Source: Evol Appl. 2015 May 28;8(6):573–85. doi: 10.1111/eva.12266 (PMC4479513; doi:10.1111/eva.12266)
Supplement: Supplementary file 3 [file eva0008-0573-sd3.docx]

| **Site** | **Copper** | **Zinc** | **Arsenic** | **Cadmium** |
| --- | --- | --- | --- | --- |
| St Erth Abs | 27.38 | 545.75 | 10.92 | 1.13 |
| St Erth Gauge | 36.74 | 533.85 | 12.27 | 1.19 |
| Relubbus | 42.68 | 630.8 | 8.82 | 1.38 |
| Godolphin | 471.15 | 2511.59 | 98.68 | 2.58 |
| Drym | 6.34 | 35.99 | 2.97 | 0.46 |
| B3303 bridge | 4.97 | 35.95 | 3.66 | 0.36 |

**Supporting Information: Figure 3.** Data from the Environment Agency (1990-2014) on concentrations of metals (µgL -1) at the sites across the River Hayle. In relation to the sites listed in Table 1 - HAY1: St Erth Abstraction (Abs) and St Erth Gauge. HAY2: Relubbus. Godolphin middle region. HAY3: Drym. HAY4: B3303 bridge.
