# Supplementary material for: Human mining activity across the ages determines the genetic structure of modern brown trout (Salmo trutta L.) populations
Source: Evol Appl. 2015 May 28;8(6):573–85. doi: 10.1111/eva.12266 (PMC4479513; doi:10.1111/eva.12266)
Supplement: Supplementary file 4 [file eva0008-0573-sd4.docx]

**Supplementary Information: Figure 4.** Relationship between genetic distance (FST/(1-FST)) and geographic distance (kilometres) for the 15 populations of brown trout, based on 99 permutations; rxy=-0.09, P=0.28, R^2^=0.0082.
