# Supplementary material for: Human mining activity across the ages determines the genetic structure of modern brown trout (Salmo trutta L.) populations
Source: Evol Appl. 2015 May 28;8(6):573–85. doi: 10.1111/eva.12266 (PMC4479513; doi:10.1111/eva.12266)
Supplement: Supplementary file 5 [file eva0008-0573-sd5.docx]

|  |  | **Catchment** | **Sub-catchment** | **Site** | **Site code** | **N** | **Coordinates** |
| --- | --- | --- | --- | --- | --- | --- | --- |
|  | **Control sites** | Camel | Allen | Trehannick | CAM1 | 49 | 50.579, -4.733 |
|  |  | Camel | Stannon Stream | Stannon | CAM2 | 44 | 50.594, -4.687 |
|  |  | Gannel | Main River | Gwills | GAN1 | 50 | 50.393, -5.058 |
|  |  | Gannel | Main River | Kestle Mill | GAN2 | 50 | 50.393, -5.024 |
|  |  | Fal | Main River | Tregony | FAL | 47 | 50.267, -4.918 |
|  |  | Tresillian | Main River | Geen Mill | TRES | 48 | 50.286, -4.981 |
|  | **Metal-impacted sites** | Red River | Tehidy Brook | Tehidy Brook | RR1 | 45 | 50.233, -5.325 |
|  |  | Red River | Main River | Roseworthy farm | RR2 | 41 | 50.20, -5.341 |
|  |  | Hayle | Main River | St Erth | HAY1 | 44 | 50.164, -5.433 |
|  |  | Hayle | Main River | Porthcollum | HAY2 | 48 | 50.149, -5.421 |
|  |  | Hayle | Main River | Drym farm | HAY3 | 48 | 50.149, -5.335 |
|  |  | Hayle | Main River | Clowance wood | HAY4 | 37 | 50.157, -5.330 |
|  |  | Coastal Streams | Crowlas | Cuccurian | CRO | 49 | 50.159, -5.494 |
|  |  | Trevaylor | Main River | Trythogga | TREV1 | 50 | 50.127, -5.526 |
|  |  | Trevaylor | Main River | Noongallas | TREV2 | 50 | 50.145, -5.551 |
|  |  |  |  |  |  |  |  |

**Supporting Information: Table 1.** Location and site identification for each sampled population. N - number of individuals sampled. Coordinates in decimal degrees (WGS84). The populations are grouped into ‘clean’ sites, where there is little contemporary metal-contamination, and ‘metal-impacted sites’, which are currently negatively affected by metal pollution. Site codes match those presented in Figure 1.
