# Supplementary material for: Human mining activity across the ages determines the genetic structure of modern brown trout (Salmo trutta L.) populations
Source: Evol Appl. 2015 May 28;8(6):573–85. doi: 10.1111/eva.12266 (PMC4479513; doi:10.1111/eva.12266)
Supplement: Supplementary file 6 [file eva0008-0573-sd6.docx]

| **Multiplex** | **Locus** | **Reference** | **F** | **R** | **Volumes** | **PCR Program** |
| --- | --- | --- | --- | --- | --- | --- |
| MP1a | Ssa85 | O’Reilly *et al.* 1996 | 1μM | 1μM | 28μM Primer | TD: 60°-50° |
|  | Ssa52NVH | Gharbi *et al.* 2006 | 8μM | 8μM | 72μM H20 |  |
|  | SsosL417 | Slettan *et al.* 1995 | 1μM | 1μM |  |  |
|  | SS11 | Martinez *et al.* 1999 | 4μM | 4μM |  |  |
| MP1b | BG935488 | Vasemägi *et al.* 2005 | 1.5μM | 1.5μM | 33μM Primer | TD: 60°-50° |
|  | CA048828 | Vasemägi *et al.* 2005 | 6μM | 6μM | 67μM H20 |  |
|  | CA060177 | Vasemägi *et al.* 2005 | 8μM | 8μM |  |  |
|  | CA060208 | Vasemägi *et al.* 2005 | 1μM | 1μM |  |  |
| MP2a | SsaD157 | King *et al.* 2005 | 4μM | 4μM | 30μM Primer | TD: 55°-45° |
|  | SsaD58 | King *et al.* 2005 | 6μM | 6μM | 70μM H20 |  |
|  | SsaF43 | Sánchez *et al.* 1996 | 1μM | 1μM |  |  |
|  | SsosL311 | Slettan *et al.* 1995 | 4μM | 4μM |  |  |
| MP2b | Str3QUB | Keenan *et al.* 2013 | 1μM | 1μM | 14μM Primer | TD: 60°-50° |
|  | Ssa407UOS | Cairney *et al.* 2000 | 6μM | 6μM | 86μM H20 |  |
| MP3a | Ssa197 | O’Reilly *et al*.1996 | 1μM | 1μM | 14μM Primer | TD: 60°-50° |
|  | sasaTAP2A* | Grimholt *et al.* 2002 | 2μM | 2μM |  |  |
| MP3b | SSsp2213 | Paterson *et al.* 2004 | 4μM | 4μM | 14μM Primer | TD: 55°-45° |
|  | One102 | Olsen *et al.* 2000 | 3μM | 3μM | 86μM H20 |  |
| MP4a | CA053293 | Vasemägi *et al.* 2005 | 8μM | 8μM | 60μM Primer | TD: 60°-50° |
|  | sasa-UBA* | Grimholt *et al.* 2002 | 6μM | 6μM | 40μM H20 |  |
|  | CA769358 | Vasemägi *et al.* 2005 | 4μM | 4μM |  |  |
|  | CA515794 | Vasemägi *et al.* 2005 | 8μM | 8μM |  |  |
|  | sasaTAP2B* | Grimholt *et al.* 2002 | 4μM | 4μM |  |  |
| MP4b | Ssa412UOS | Cairney *et al.* 2000 | 5μM | 5μM | 10μM Primer | TD: 60°-50° |

**Supporting Information. Table 2:** Details of the eight multiplexes including quantities of forward and reverse primer, total volumes and PCR touchdown (TD) programme. For each numerically identified multiplex, groups a and b were pooled together for analysis on the Beckman Coulter sequencer. *MHC class 1 linked loci
