# Supplementary material for: Human mining activity across the ages determines the genetic structure of modern brown trout (Salmo trutta L.) populations
Source: Evol Appl. 2015 May 28;8(6):573–85. doi: 10.1111/eva.12266 (PMC4479513; doi:10.1111/eva.12266)
Supplement: Supplementary file 7 [file eva0008-0573-sd7.docx]

| CAM2 | GAN1 | GAN2 | FAL | TRES | RR1 | RR2 | HAY1 | HAY2 | HAY3 | HAY4 | CRO | TREV1 | TREV2 |  |
| --- | --- | --- | --- | --- | --- | --- | --- | --- | --- | --- | --- | --- | --- | --- |
| *** | *** | *** | *** | *** | *** | *** | *** | *** | *** | *** | *** | *** | *** | CAM1 |
| 0.000 | *** | *** | *** | *** | *** | *** | *** | *** | *** | *** | *** | *** | *** | CAM2 |
| 0.022 | 0.000 | * | *** | *** | *** | *** | *** | *** | *** | *** | *** | *** | *** | GAN1 |
| 0.023 | 0.007 | 0.000 | *** | *** | *** | *** | *** | *** | *** | *** | *** | *** | *** | GAN2 |
| 0.019 | 0.027 | 0.031 | 0.000 | *** | *** | *** | *** | *** | *** | *** | *** | *** | *** | FAL |
| 0.020 | 0.024 | 0.028 | 0.014 | 0.000 | *** | *** | *** | *** | *** | *** | *** | *** | *** | TRES |
| 0.045 | 0.046 | 0.047 | 0.050 | 0.045 | 0.000 | *** | *** | *** | *** | *** | *** | *** | *** | RR1 |
| 0.039 | 0.040 | 0.038 | 0.043 | 0.045 | 0.030 | 0.000 | *** | *** | *** | *** | *** | *** | *** | RR2 |
| 0.071 | 0.073 | 0.071 | 0.077 | 0.081 | 0.093 | 0.073 | 0.000 | 0.602 | *** | *** | *** | *** | *** | HAY1 |
| 0.073 | 0.073 | 0.073 | 0.081 | 0.084 | 0.093 | 0.075 | 0.006 | 0.000 | *** | *** | *** | *** | *** | HAY2 |
| 0.057 | 0.055 | 0.057 | 0.060 | 0.060 | 0.071 | 0.058 | 0.024 | 0.026 | 0.000 | ** | *** | *** | *** | HAY3 |
| 0.063 | 0.061 | 0.062 | 0.069 | 0.066 | 0.076 | 0.063 | 0.027 | 0.029 | 0.012 | 0.000 | *** | *** | *** | HAY4 |
| 0.060 | 0.071 | 0.066 | 0.063 | 0.063 | 0.073 | 0.060 | 0.103 | 0.106 | 0.084 | 0.094 | 0.000 | *** | *** | CRO |
| 0.036 | 0.040 | 0.039 | 0.038 | 0.037 | 0.055 | 0.047 | 0.082 | 0.086 | 0.062 | 0.069 | 0.048 | 0.000 | *** | TREV1 |
| 0.059 | 0.058 | 0.058 | 0.060 | 0.056 | 0.069 | 0.068 | 0.099 | 0.101 | 0.077 | 0.080 | 0.063 | 0.022 | 0.000 | TREV2 |

**Supporting Information: Table S3.** Pairwise F_ST_ results for each of the 15 brown trout populations. F_ST_ values lie below the black diagonal boxes, significance values are above the black diagonal boxes: * = p<0.05, ** = p<0.01, *** = p<0.001.
